# Remodeling of Cellular Respiration and Insulin Signaling Are Part of a Shared Stress Response in Divergent Bee Species

**DOI:** 10.3390/insects16030300

**Published:** 2025-03-13

**Authors:** Nicole C. Rondeau, Joanna Raup-Collado, Helen V. Kogan, Rachel Cho, Natalie Lovinger, Fatoumata Wague, Allison J. Lopatkin, Noelle G. Texeira, Melissa E. Flores, David Rovnyak, Jonathan W. Snow

**Affiliations:** 1 Biology Department, Barnard College, New York, NY 10027, USA; ncrondeau@gmail.com (N.C.R.); hvk2105@columbia.edu (H.V.K.); flores39@illinois.edu (M.E.F.); 2 Department of Chemistry, Bucknell University, Lewisburg, PA 17837, USA; jrc061@bucknell.edu (J.R.-C.); drovnyak@bucknell.edu (D.R.); 3 Department of Chemical Engineering, University of Rochester, Rochester, NY 14642, USA; allison.lopatkin@rochester.edu

**Keywords:** honey bee, leafcutting bee, heat shock response, lactate dehydrogenase, cellular stress response

## Abstract

The honey bee (*Apis mellifera*) is of paramount importance to human activities through the pollination services they provide in agricultural settings. Honey bee colonies in the United States have suffered from an increased rate of annual die-off in recent years, stemming from a complex set of interacting stressors that remain poorly described. Defining the cellular responses that are perturbed by divergent stressors represents a key step in understanding these synergies. We found that multiple model stressors induce upregulated expression of the *lactate dehydrogenase* (*Ldh*) gene in the midgut of the eusocial honey bee and that the *Ldh* gene family is expanded in diverse bee species. Alterations in *Ldh* expression were concomitant with changes in the expression of other genes involved in cellular respiration and genes encoding insulin/insulin-like growth factor signaling (IIS) pathway components. Additionally, changes in metabolites in the midgut after stress, including increased levels of lactate, linked metabolic changes with the observed changes in gene expression. Select transcriptional changes in response to stress were similarly observed in the solitary alfalfa leafcutting bee (*Megachile rotundata*). Thus, increased *Ldh* expression may be part of a core stress response remodeling cellular respiration and insulin signaling. These findings suggest that a conserved cellular response that regulates metabolic demands under diverse stressful conditions may play a protective role in bees regardless of life history.

## Simple Summary

Honey bees are critical pollinators in agriculture. They have been experiencing increased mortality in the last few decades due to multiple stressors, ranging from decreased diversity of resources for foraging resulting in nutritional stress to increased pathogenicity of viruses. However, we do not yet fully understand how these stressors interact and impact honey bee health, nor how honey bees respond to these complex and often intertwined challenges. We also lack reliable biomarkers that detect cellular stress or dysfunction in honey bees at the individual or colony levels. Using real-world and model abiotic stressors, we quantified changes in the expression of a specific gene that appears to function as a metabolic switch in response to stress in eusocial honey bees. Changes in the expression of this and other metabolic genes correlated with changes in metabolites, guiding a model in which varied abiotic challenges lead to alterations in central metabolism potentially orchestrated by the insect hormone insulin-like peptide. We further found that some of these changes are found in the solitary alfalfa leafcutting bee, suggesting that bees with divergent life histories may similarly rely on cellular strategies that alter metabolism in response to stress.

## 1. Introduction

Honey bees (*Apis mellifera*) supply pollination services of vital importance to humans in primarily agricultural settings [1]. These critical pollinators have recently suffered from reduced colony level survival, caused by complex set of interacting stresses [2]. Stressors implicated in honey bee disease include nutritional stress due to loss of appropriate forage, chemical poisoning from pesticides, changes in natural living conditions brought about through large-scale beekeeping practices, myriad environmental changes due to climate change, and infection by insect parasites and pathogenic microbes [3].

Disparate stressors, including those believed to contribute to honey bee colony losses, are known to disrupt proteostasis. The homeostasis of protein synthesis, folding, function, and degradation as it occurs at both the cellular and organismal levels [4] is maintained by responses of the proteostatic network [5]. We recently used an unbiased approach to identify novel genes induced in honey bees by exposure to diverse stressors that represented model and real-world insults of abiotic origin. After subjecting adult honey bee workers to thermal stress, endoplasmic reticulum (ER) stress, or ribosomal stress, we used transcriptomic profiling (RNASeq) of the midgut to identify differences in gene expression [6,7,8]. We hypothesized that we could use these data sets to generate a set of overlapping target genes that are induced by diverse abiotic stressors with the goal of identifying candidate biomarkers that may signal poor colony health.

Eusocial honey bees live in large colonies comprising three castes, which allows them to decouple reproductive labor from all other habitat maintenance tasks [9]. Additionally, they exhibit a unique division of labor that maximizes the efficiency of the colony by separating the responsibilities of brood care from hive maintenance and the acquisition of resources [10]. While social living increases the likelihood of encountering a diversity of stressors through exposure by proximity of cohabitants alone, it also has allowed honey bees to develop increased resilience against some of these stressors through social behaviors to combat biotic (reviewed in [11]) and abiotic stressors [12]. In addition to understanding how honey bees are affected by and respond to various stressors, it is critical that we further define the factors that influence non-*Apis* bee species, including those with solitary lifestyles, whose populations are facing sharp reductions [13]. Currently, it is thought that many of the stressors exacerbating honey bee colony losses also affect the health of native, solitary bee species. Thus, we further hypothesized that the same suite of cellular stress genes identified in our honey bee data sets would be conserved in bee species with divergent life histories by comparing the expression of these genes in honey bees with expression in the alfalfa leafcutting bee (*Megachile rotundata*).

## 2. Materials and Methods

### 2.1. Honey Bee Caging, Treatments, and Tissue Collection

The study complied with relevant institutional, national, and international guidelines and legislation. Unless otherwise stated, honey bees were collected from the landing board of outbred colonies in New York, New York, consisting of a typical mix of *Apis mellifera* subspecies found in North America at different times during the months of April–October. Only visibly healthy honey bees were collected and all source colonies were visually inspected for symptoms of common bacterial, fungal, and viral diseases of honey bees (Nosemosis, foulbrood, chalkbrood, stonebrood, sacbrood, and deformed wing symptoms), as well as Varroa mite infestation, during biweekly hive checks. Colonies were treated for Varroa mite infestation using ApiGuard and *Vairimorpha* infection using Fumagillin, both according to the manufacturers’ instructions during the spring build up. Frames of bees hatched in incubators were similarly monitored for these diseases. For experiments analyzing diverse stressors, adult worker bees were collected from the landing board and kept in 2.2 × 8.6 × 21.3 cm acrylic cages with sliding doors machined at Carleton Labs, Columbia University. For these experiments, bees were fed via a modified 1.5 mL screw-cap tube containing 33% sucrose solution that in cases of chemical stressors was supplemented with a chemical treatment. When used, newly eclosed honey bees were collected after emerging from a capped brood frame overnight maintained in an incubator at 35 °C. Approximately 30 newly eclosed bees were placed in each acrylic cage. All caged honey bees were maintained in incubators at 35 °C (unless otherwise stated) at a relative humidity between 50 and 60% in the presence of PseudoQueen. For the chemical mediated-stress trials, sucrose feeders were supplemented with 1 mM paraquat (oxidative), 24 µM tunicamycin (ER), 200 µM halofuginone (tRNA synthetase), 200 µM ixazomib (proteasome), 25 µg/mL bleomycin (DNA damage), or 0.5 mg/mL cycloheximide (ribosome) for 24 or 48 h. These compounds and their effects have been characterized in *Drosophila melanogaster* [14,15,16,17] and honey bees [6,7,18,19], with the exceptions of halofuginone and bleomycin, which have solely been used in honey bees [7,20]), [21], and ixazomib, for which the honey bee effects have not yet been published (Snow, unpublished observations). For thermal stress trials, honey bees were maintained for four hours in cages at 35° or heat-shocked at 45 °C [21]. After cold anesthesia, the following tissues were dissected for gene expression analysis: head tissue (predominantly brain, hypopharyngeal gland, and mandibular gland), midgut, thorax, and abdominal wall. All dissected tissues were placed in RNAlater (Invitrogen, San Diego, CA, USA) for storage prior to RNA extraction.

### 2.2. Alfalfa Leafcutting Bee Adult Caging and Tissue Collection

As described previously [22], 4–6 eclosed alfalfa leafcutting bee (*Megachile rotundata*) females were placed in the cages described above using an InsectaVac Aspirator (Bioquip, Rancho Dominguez, CA, USA). Cages were kept in incubators maintained on a 12 h light, 12 h dark cycle, at 30 °C. Survival was recorded daily, and dead alfalfa leafcutting bees were removed. Each cage was provided with one modified 1.5 mL Eppendorf tube with 30% sucrose solution so that alfalfa leafcutting bees could feed ad libitum, with the sucrose solution tube replaced once every 2–3 days.

### 2.3. RNA Isolation, Reverse-Transcription and Quantitative PCR for Gene Expression Analysis

RNA was prepared from the relevant tissue of the bees, as previously described [19,21]. Tissues were manually crushed with a disposable pestle in Trizol Reagent (Invitrogen, San Diego, CA, USA) and RNA was then extracted as per the manufacturer’s instructions. RNA was then DNase I-treated by RQ1 RNase-Free DNase (Promega, Madison, WI, Canada) and cDNA was synthesized using approximately 1 μg of RNA and the High-Capacity cDNA Reverse Transcription Kit with RNase Inhibitor (Applied Biosystems, Foster City, CA, USA) [19,21]. Additionally, 1 μL of cDNA was used as a template in conjunction with PowerUp SYBR Green Master Mix (Applied Biosystems, Foster City, CA, USA) and appropriate primers for quantitative PCR (qPCR). Reactions were run in a LightCycler 480 thermal cycler (Basel, Switzerland) or Bio-Rad CFX Opus (Bio-Rad, Hercules, CA, USA). The PCR conditions were as follows: 94 °C for 2 min, followed by 94 °C for 15 s, 60 °C for 30 s, and 72 °C for 60 s for 40 cycles. These steps were followed by a 10 min extension step at 72 °C [19,21]. The primer sequences targeting the transcripts of the genes of interest can be found in Supplementary Table S1. The difference between the threshold cycle (Ct) number for β-actin and that of the gene of interest was used to calculate the level of that gene relative to β-actin using the 2^(−ΔCT)^ method [23]. All qPCR data represent the expression values from individual bees (sample sizes found in figure legends) and is displayed as the mean ± SEM.

### 2.4. RNA-Seq

For RNA-Seq analysis, we used data sets generated previously [6,7,8]. Briefly, we performed transcriptome profiling (RNASeq) on midguts from bees: (1) fed sucrose solution containing 24 μM tunicamycin or DMSO for 24 h, (2) maintained at either 35 or 45 °C for 4 h, or (3) treated with sucrose solution containing 200 µM halofuginone or DMSO for 24 h. RNASeq analysis was performed on 3 midguts from each group individually. Libraries were prepared using the NEBNext Ultra RNA library preparation kit and then sequenced using the paired end 150 bp sequencing configuration on the Illumina HiSeq 4000 platform. After the sequence reads were trimmed, they were then mapped to the *Apis mellifera* reference genome then available on NCBI (Amel 4.5 version). After mapping and total gene hit counts calculation, the total gene hit counts table was used for downstream differential expression analysis using DESeq2. The original *p*-values are generated using the Wald test. The adjusted/corrected *p*-values are obtained using the Benjamini and Hochberg method. Genes with adjusted *p*-value < 0.05 and Absolute Log2Fold Change > 1 were called as significant differentially expressed genes for each comparison. The RNA sequence information in this study was previously submitted to the Gene Expression Omnibus database under the accession numbers GSE139368, GSE159083, and GSE165411 (https://www.ncbi.nlm.nih.gov/). Venn diagram analysis of gene lists from these RNA-Seq data was performed using the following webtool (http://bioinformatics.psb.ugent.be/webtools/Venn/ accessed on 26 February 2025), as described previously [19].

### 2.5. Nuclear Magnetic Resonance (NMR) Metabolite Quantification

Excised midguts were stored in individual microcentrifuge tubes (−78 °C, dry ice) for transport and subsequently stored at −80 °C. A recently determined optimal aqueous metabolite extraction protocol for diverse honey bee samples (brain, head, body, whole bee) was used here [24]. To each tube, 1 mL of a 2:1 *V*/*V* solution of acetonitrile/water was added along with two steel beads (1/8” and 1/16”). A reciprocal bead-beating apparatus was used for thorough homogenization followed by high-speed centrifugation (10 min, 18 k rcf) to pelletize cell debris and precipitate biomacromolecules. The resulting supernatant was isolated and subjected to rotary vacuum centrifugation (3 h, 1 torr, no heating), resulting in small dry pellets that were frozen at −80 °C. Each pellet was resuspended with vortex agitation in 350 μL of NMR Buffer (99% D_2_O, 0.1 mM TSP (IUPAC: sodium 3-trimethylsilylpropionate), 100 mM phosphate, pH 7.4) and transferred to a Shigemi^TM^ susceptibility-matched NMR tube. Due to small broad background (residual protein/lipid) signals, one-dimensional presaturation-CPMG (100 cycles, 1 ms interpulse delay) ^1^H NMR spectra were acquired (352 transients, 8 steady state scans, 3 s presaturation, 4 s acquisition, 7 s recycle, t(π/2) = 7.6 µs, 66 min each). All spectra were obtained on a 14.1 T spectrometer (600 MHz for ^1^H, Varian Inc., Palo Alto, CA, USA, DDR1/VNMRS generation console, vnmrj 4.2) using an inverse triple resonance probe. Spectra were profiled manually by J.R.C. and D.S.R. using the Chenomx NMR Suite 8.1 (Chenomx, Edmonton, AB, Canada) and are reported as concentrations (sample volume 0.350 mL). In order to clearly distinguish lactate from threonine, each sample was shimmed to an initial TSP line width of 0.8 Hz or lower. Analysis employed line broadening (0.3 Hz), phasing, baseline correction (spline), and reference deconvolution of the 0 ppm TSP peak. Profiling included a limited number of putative singlets at well-conserved spectral positions.

### 2.6. Identification and Analysis of Putative LDH Proteins in Bee (Hymenoptera: Apoidea: Anthophila) Genomes

For a complete list of genes encoding LDH proteins in bee genomes, we used each honey bee protein coding sequence as the query to search representative bee genomes for which non-redundant sequences are available in addition to the *D. melanogaster* LDH-encoding genes (NCBI Gene IDs: 45880 and 36510) and the *Homo sapiens L-lactate dehydrogenase A-like 6A* gene (LDHAL6A, NCBI Gene ID: 160287). We also used the honey bee protein sequences to identify LDH-encoding genes in the genomes of insects of other orders and other insects within Hymenoptera. For tree generation, protein alignments were generated with MUSCLE [25] using default parameters and inspected manually. A maximum-likelihood phylogenetic tree was inferred from the resulting alignment using the RaxML program version 8 [26] with the GAMMA model. Bootstrapping was conducted with 100 replicates. The resulting tree was visualized and annotated using ggtree in R. LDH active sites were predicted using https://prosite.expasy.org/ (accessed on 10 February 2021) as defined by the pattern PS00064, ([LIVMA]-G-[EQ]-H-G-[DN]-[ST]).

### 2.7. Statistical Analysis

Using GraphPad Prism (v10.4.1), data were log10 transformed and compared using unpaired t-tests with Welch’s correction when values fit normal distributions or Mann–Whitney U non-parametric tests when they did not fit normal distributions. Normality was assessed using Shapiro–Wilk tests. When more than two groups were being compared (Supplementary Figure S2D), data were compared using one-way ANOVA with Tukey’s multiple comparison tests when values fit normal distributions or Kruskall–Wallis tests. For survival analysis (Supplementary Figure S3A), treated versus untreated groups were compared using the Mantel–Cox test.

In the statistical treatment of targeted NMR metabolomic data, IBM SPSS 28.0.1.0 (142) was used, all outliers were retained, and there were no missing values. When comparing metabolite concentrations of the control and halofuginone groups, an independent samples *t*-test was performed; however, the Mann–Whitney U test was reported if Shapiro–Wilk was significant, while Welch’s test was reported if Levene’s test for homogeneity of variances was significant. Cluster (e.g., PLSDA) and additional (e.g., correlation, AUROC) multivariate analysis of targeted NMR metabolomic data was performed in the MetaboAnalyst environment [27,28]. In MetaboAnalyst, concentrations were log and Pareto scaled; however, observed trends were generally insensitive to specific choices of scaling. Detailed descriptions of the statistical analysis used for the figures can be found in Supplementary Table S2.

## 3. Results

### 3.1. Ldh Identified in Screen for Shared Stress Response Genes in Honey Bees

Examining the published transcriptomics data sets [6,7,8], we observed upregulation of genes encoding L-lactate dehydrogenase (LDH) proteins, which catalyze the interconversion of pyruvate and lactic acid, in every data set. Examination of genes encoding LDH proteins in honey bees (Hymenoptera), showed that this species possesses four genes with predicted lactate dehydrogenase activity rather than two as expected from studies in fruit flies (Diptera) (Supplementary Table S3). Comparison of the sequences of these genes revealed that insects from other orders had either one or two genes predicted to encode LDH proteins (Supplementary Table S3). Within Hymenoptera, we found examples of wasps, ants, and sawflies with three or four genes encoding LDH proteins (Supplementary Table S3). In honey bees and other bees examined, these genes are located in a single cluster in the genome and share a similar gene order (Supplementary Figure S1, Supplementary Table S4). Transcripts for one of these genes, *LOC411888,* were significantly upregulated by treatments activating the Heat Shock Response (HSR) and Integrated Stress Response (ISR) through thermal and ribosomal stress, while transcripts for another, *LOC411887*, were increased by Unfolded Protein Response (UPR) induction through ER stress.

**Figure 1:**
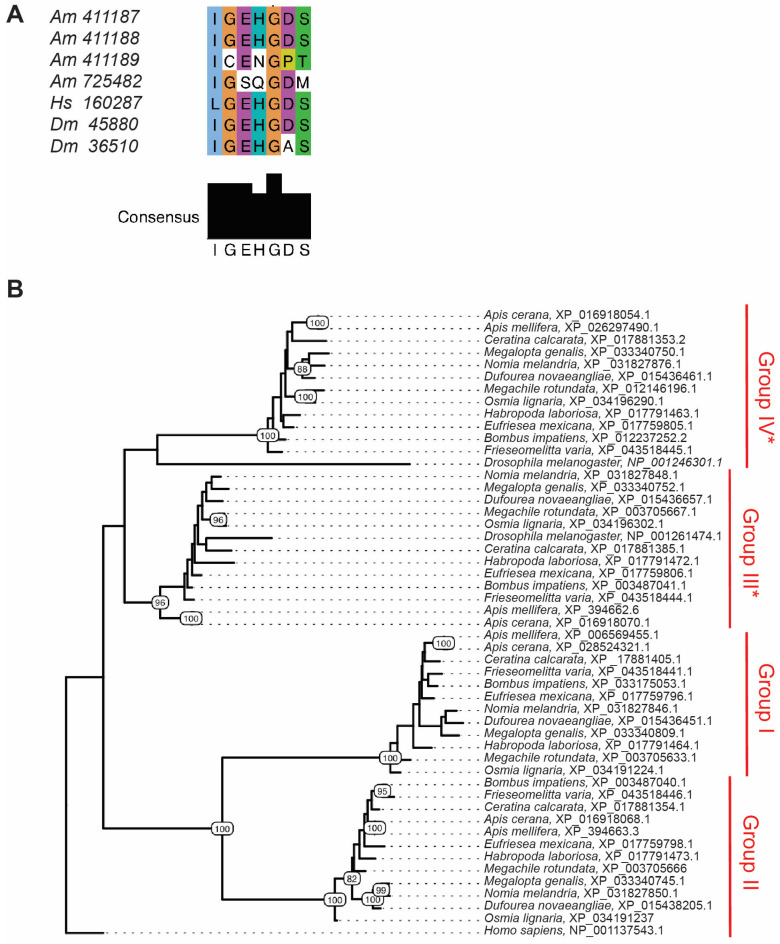
Expanded putative LDH-like proteins are shared among bees. The predicted active sites (or homologous region) for the honey bee LDH proteins encoded by 411188 (193–199), 411187 (272–278), 411189 (201–208), and 725482 (214–221), for the *H. sapiens* LDH protein encoded by 160287 (190–196), and for the *D. melanogaster* LDH protein encoded by 45880 (190–196) and 36510 (218–225) (**A**). Phylogenetic tree of bee LDH proteins, *D. melanogaster* LDH proteins, and *H. sapiens* LDHA-like 6A protein based on full amino acid sequences. Group I–IV represents the order of the encoding genes in the genomes of the respective species and * denotes the groups containing the key active site Histidine. Scale bar represents the number of amino acid changes per site (**B**).

Examination of *LOC411888* levels in the UPR data set revealed that while this gene was induced in this data set, the presence of a high outlier precluded significance with the limited sample size used for RNAseq (n = 3 midguts for each condition) (Supplementary Tables S4 and S5). The *LOC411888 Ldh* gene encodes the protein most homologous to *Drosophila* LDH *encoded* by the *Ecdysone-inducible gene L3* (*Impl3*) gene. Of the other three *Ldh*-like genes, *LOC411187* (described above), is also predicted to possess lactate dehydrogenase activity and was upregulated in the UPR data set, but no others (Supplementary Table S6). Interestingly, both the *LOC411189* and *LOC725482* genes encode proteins that share significant homology with other LDH-encoding genes but do not contain the key histidine residue in the active site, suggesting that they may not be able to catalyze the pyruvate/lactate reaction (Figure 1A, Supplementary Table S4).

To explore conservation of these proteins between bee species and to better understand their relationship to the two LDH proteins from *D. melanogaster*, we searched for genes encoding LDH homologs in other bee genomes using the amino acid sequences of the four honey bee LDH proteins, and constructed a phylogenetic tree. These genomes represent both social and solitary bee species broadly distributed phylogenetically within bees. The LDH protein from *H. sapiens* was used as an outgroup. As expected, we found that the bee LDH proteins cluster as four groups (Figure 1B, Supplementary Table S4) that match their location in the *Ldh* locus in the same gene order found for *A. mellifera* (e.g., see *M. rotundata*, Supplementary Figure S1A). Similarly, other bee species possess two LDH proteins predicted to have intact active sites and two predicted to be catalytically inactive. (e.g., see *M. rotundata*, Supplementary Figure S1B and Supplementary Table S4).

### 3.2. Additional Types of Cellular Stress Increase Ldh Expression in Honey Bees

**Figure 2:**
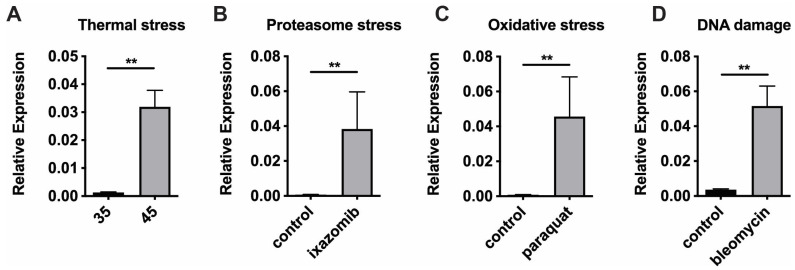
*Ldh* is induced by diverse stressors. Transcript levels of the *Ldh (Impl3)* relative to *β-actin* in midgut tissue from adult worker bees captured at the landing board after thermal stress (with bees maintained for four hours in cages at either 45 °C or the control temperature 35 °C) (**A**), or after pharmacological induction of proteasome stress (**B**), oxidative stress (**C**), or DNA damage (**D**). Mean ± SEM is shown and represents expression values of the genes of interest calculated using the 2^(−ΔCT)^ method for individual bees. Statistical significance is noted as **, *p* < 0.01.

We previously demonstrated that ISR induction by tRNA synthetase inhibition via halofuginone, ribosome inhibition by cycloheximide, and tunicamycin-induced ER stress [7] upregulates the expression of *Ldh* (Supplementary Figure S2A). To recapitulate these findings in response to other chemical stressors, we first examined heat-shock dependent induction of *Ldh* in midgut tissues from honey bees kept at either at 35° or 45 °C for 4 h using qPCR. We observed significantly increased levels of *Ldh* transcripts after heat shock (Figure 2A).

Proteasome inhibition (via ixazomib), oxidative stress induction (via paraquat), and DNA damage (via bleomycin) all significantly increased expression of *Ldh* in the midguts of honey bees as well (Figure 2B–D). Interestingly, we found that none of the other three *Ldh-like* genes (*LOC411187*, *LOC411189*, and *LOC725482*) were upregulated after thermal stress, tRNA synthetase inhibition, or ER stress (unpublished observations).

### 3.3. Ldh Expression Is Induced in Multiple Tissues by Thermal Stress

**Figure 3:**
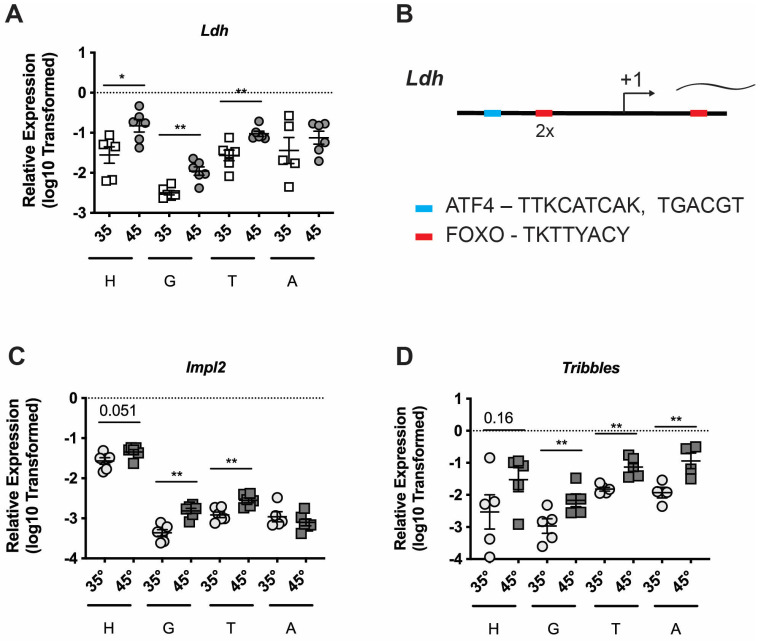
Thermal stress induces *Ldh* and insulin signaling components in multiple tissues. Transcript levels of *Ldh* relative to *β-actin* in head tissue (H), midgut (M), thorax tissue (T), and abdominal wall (**A**) from adult worker bees captured at the landing board and maintained for four hours in cages at either 35° or 45 °C (**A**). Schematic of *Ldh* promoter region (−2 kb to +1 kb relative to the transcriptional start site) (**B**). Transcript levels of *ImpL2* (**C**) *and Tribbles* (**D**) relative to *β-actin* for the tissues above. Symbols represent expression values of the genes of interest calculated using the 2^(−ΔCT)^ method for individual bees. Mean ± SEM is also shown. Statistical significance is noted as *, *p* < 0.05, and **, *p* < 0.01.

While orally administered treatments appear to reliably affect only the digestive tract, we can apply thermal stress to all tissues equally. Thus, we examined *Ldh* expression in multiple tissues, including head tissue (predominantly brain, hypopharyngeal gland, and mandibular gland), midgut, thorax, and abdominal wall from adult worker bees caged at 35 °C or heat shocked at 45 °C for 4 h (Figure 3A). We observed significant induction of *Ldh* in all tissues but the abdomen, suggesting that upregulation of *Ldh* is part of a cellular stress response across multiple tissue types. Interestingly, insect fat bodies function as nutrient reserves.

We also took advantage of a previously collected sample set for which RNA had been extracted using the same methods described above from the same set of tissues for sterile attendant workers and reproductive queens [29]. Caged individuals were maintained at 35 °C or 45 °C for 4 h to determine whether reproductive potential impacts upregulation of *Ldh* following heat shock. We found that *Ldh* expression increased in queen midguts to a degree comparable to that of workers in response to thermal stress (Supplementary Figure S2B). While the other *Ldh*-like genes (*LOC411187*, *LOC411189*, and *LOC725482*) were expressed at similar levels in control queen and worker midguts of honey bees caged at 35 °C, these three genes were not induced after thermal stress (unpublished observations).

### 3.4. Broader Transcriptional Remodeling of Cellular Respiration and Insulin Signaling Are Altered as Part of a Shared Stress Response in Honey Bees

Based on the diversity of stresses demonstrated to promote gene induction, we were interested in exploring the potential *trans* factors and *cis* elements responsible for regulating increased gene expression. In the fruit fly, *Ldh* is increased after ER stress, which also results in upregulation of glycolysis-associated genes and downregulation of both citric acid cycle (TCA)- and oxidative phosphorylation-associated genes, suggesting a significant remodeling of metabolic pathways [30]. However, we did not observe changes in the expression of genes involved in glycolysis, TCA, or oxidative phosphorylation that followed these trends. In fact, we instead observed that *mitochondrial cytochrome C* (*CytC*, NCBI Gene ID *408270*) and *succinate dehydrogenase [ubiquinone] flavoprotein subunit*, *mitochondrial-like* (*SdhAl*, NCBI Gene ID *408734*), genes encoding proteins involved in the mitochondrial electron transport chain, were upregulated 2-fold to 3-fold in all RNASeq sets (Supplementary Table S6) [6,7,8], while other genes involved in glycolysis, TCA, or oxidative phosphorylation were unchanged (unpublished observations).

Lee et al. [30] found that binding sites upstream from the *Ldh* transcriptional start site of the UPR transcription factor Activating Transcription Factor 4 (ATF4), which regulates targets through cyclic AMP response elements (CRE) (TGACGT, TTKCATCAK), are critical for gene induction in luciferase reporter systems. We found evidence of one such ATF4-binding motif (Figure 3B, Supplementary Figure S3). We then looked at other sites known to mediate responses to the stresses triggered above (Supplementary Figure S3). We did not find evidence of canonical Heat Shock Elements (HSEs, consensus sequence = GAANNTTCNNGAA [31,32]) in the *Ldh* promoter.

Next, we examined the promoter regions for evidence of direct regulation by UPR-activated transcription factors. Three *cis*-acting response elements have been characterized in the promoter regions of UPR-induced genes in mammals. These are the ERSE (ER Stress Response Element, CCAATN9CCACG), the ERSE-II (ER Stress Response Element II, ATTGGNCCACG), and the UPRE (Unfolded Protein Response Element, TGACGTGR) (reviewed in [33]). The transcriptional regulator controlling the oxidative stress response, NRF2 (and its homologs), is responsible for promoting resistance to oxidative stress in *Caenorhabditis elegans*, *D. melanogaster*, and mammals (reviewed in [15]). Nrf2 responsive genes possess the consensus binding site (TGAYNNNGC) that makes up the core of the antioxidant responsive element (ARE). We did not find evidence of an ARE in the *Ldh* promoter. Finally, the transcription factor FOXO has been shown to regulate *Ldh* in *D. melanogaster* [34]. We observed consensus sites for FOXO binding (TKTTYACY [35]) in the *Ldh* promoter (Figure 3B, Supplementary Figure S3). Thus, our data suggest that *Ldh* could be regulated by ATF4 and FOXO, potentially acting in concert. The *SdhAl* gene also possesses potential binding sites for FOXO (but not ATF4) (Supplementary Figure S3).

FOXO is the key transcription factor involved in the insulin/insulin-like growth factor signaling (IIS) pathway that responds to both nutrient levels and stress [36,37]. The data above suggest that the IIS pathway may be critical for the transcriptional changes associated with cellular respiration. We examined the previously mentioned RNAseq data sets for genes that might indicate changes in IIS and observed that *ecdysone-inducible gene L2* (*Impl2*) and *tribbles* (*Trbl*) are both increased in honey bee midguts upon all three treatments [6,7,8] (Supplementary Table S6). *ImpL2* is a homolog of insulin-like growth factor binding proteins in mammals [38]. It binds insulin-like peptides and inhibits their interaction with the insulin receptor and has been implicated in inter-organ communication and modulation in *D. melanogaster* [39,40]. *Tribbles* is another insulin-responsive gene first discovered in the fruit fly [41]. It encodes a pseudokinase that has been shown to be a negative regulator of insulin signaling in mammals [42] and *D. melanogaster* [43].

We looked at the transcript levels of these two genes in response to thermal stress using qPCR and found that both *Impl2* and *Tribbles* levels were increased in honey bees exposed to 45 °C when compared to those maintained at 35 °C (Figure 3C,D). Interestingly, we found significant induction of *Impl2* in all tissues but the abdomen and *Tribbles* across all tissues, suggesting that local inhibition of insulin signaling after stress exposure may also be shared across multiple tissue types. Further, we observed consensus sites for both ATF4 and FOXO binding in the *Impl2* and *Trbl* gene promoters (Supplementary Figure S3), supporting a model in which these two genes are regulated by these transcription factors.

### 3.5. Changes in Metabolome Correlate with Transcriptional Changes in Cellular Respiration

**Figure 4:**
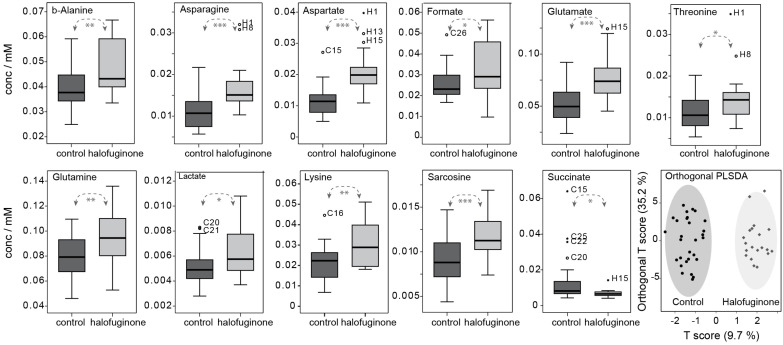
Increased lactate and certain amino acids correlate with increased *Ldh* expression. Specifically, box and whisker plots are shown for the midgut metabolite concentrations that exhibited significant *p*-values between the control and the halofuginone-treated honey bee workers. Concentrations are relative to a 350 µL volume (* *p* < 0.05, ** *p* < 0.01, *** *p* < 0.001). In the final panel, clustering of groups is shown by orthogonal PLSDA (MetaboAnalyst 5.0), where variables with VIP > 1.2 are aspartate (1.98), glutamate (1.85), asparagine (1.75), sarcosine (1.62), lysine (1.35), succinate (1.34), *β*-alanine (1.30), glutamine (1.28), lactate (1.24), and threonine (1.24). Additional PLSDA models perform well, including a five-variable sparse PLSDA model, and are provided together with a correlation plot in the Supplemental Materials.

Increased *Ldh* mRNA levels would be expected to impact levels of key metabolites involved in the reaction it catalyzes, including lactate, pyruvate, and NAD+ (Supplementary Figure S8). We used an NMR strategy for sampling the aqueous metabolome, which has been pursued in honey bees previously [24,44], to examine the levels of these and other metabolites in response to halofuginone feeding, which strongly induced *Ldh* expression. Despite the small sample size of the midgut, 30 aqueous metabolites were measured and quantified by NMR in each midgut (Supplementary Table S7). We confirmed that the effects of halofuginone on honey bee survival, *Ldh* induction, and stress gene induction were similar to that observed previously for older honey bees (Supplementary Figure S4 and [7]). We observed that halofuginone feeding resulted in increased levels of midgut lactate (*p* = 0.017, Figure 4 and Supplementary Table S7). Surprisingly, no significant changes occurred in pyruvate or NAD+ levels. These two metabolites are involved in numerous major pathways, which may keep their levels relatively constant.

Interestingly, we also found that the amino acid sarcosine was increased [22]. Sarcosine is a product of the reaction catalyzed by the glycine-N-methyltransferase enzyme (GNMT) in the methionine cycle, which we previously reported as another stress-induced target gene [22]. In addition, we found significant increases in some non-essential amino acids that have biosynthetic pathways branching off from the TCA cycle (asparagine, aspartate, glutamate, and glutamine), and in essential amino acids (threonine and lysine) that are synthesized in bacteria from aspartate. Neither alanine nor the branched chain amino acids (i.e., ILV) changed significantly (Figure 4 and Supplementary Table S7). We also observed a marked decrease in succinate, a key TCA metabolite. Succinate is a reactant for the enzyme succinate dehydrogenase, which we find is transcriptionally upregulated in our shared stress gene set (see above). Remodeling of the TCA cycle in the midgut in response to halofuginone treatment is supported by these findings, although the particularly strong increase in aspartate observed here could suggest a more specific organismal response may be taking place.

Finally, *β*-alanine is a product of aspartate decarboxylation in some bacteria [45,46,47]. The increase in *β*-alanine with aspartate (Pearson coefficient = 0.683, *p* < 0.001), along with the increase in lysine and threonine, may indicate that bacterial metabolism is responding to the elevated somatic aspartate levels. Cluster analysis by orthogonal PLSDA separates the groups well on the basis of metabolites that are consistent with those reported in Supplementary Table S7 (see Figure 4 caption). Additional PLSDA (Supplementary Figure S5), correlation (Supplementary Figure S6), and volcano plots (Supplementary Figure S7) are given in the Supporting Information.

### 3.6. Stress Activates the Ldh Gene in the Alfalfa Leafcutting Bee

**Figure 5:**
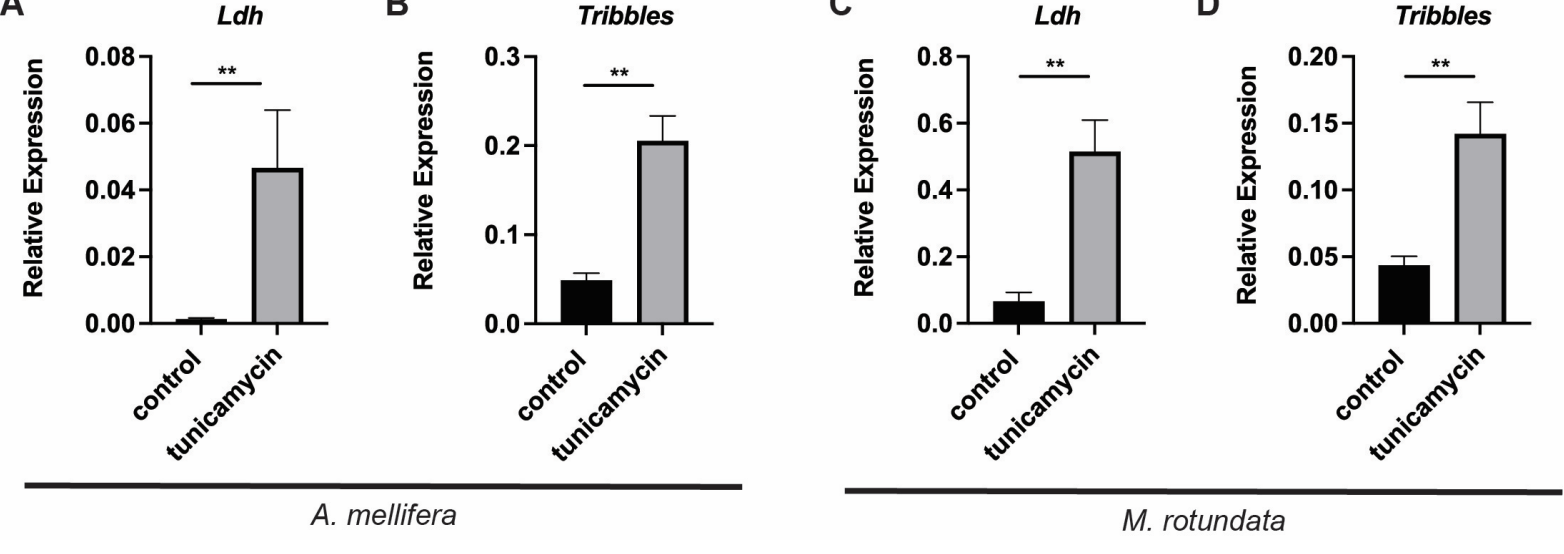
Alfalfa leafcutting bee *Ldh* is induced by tunicamycin. Transcript levels of *Ldh* (**A**) and *Tribbles* (**B**) relative to *β-actin* in midgut tissue from individual honey bees treated with 30 μM tunicamycin or a carrier control after 48 h. Transcript levels of *Ldh* (**C**) and *Tribbles* (**D**) relative to *β-actin* in midgut tissue from individual alfalfa leafcutting bees treated with 30 μM tunicamycin (T, n = 7) or a carrier control (D, n = 3) after 48 h. The difference between the threshold cycle number for *β-actin* and that of the gene of interest was used to calculate the level of that gene relative to *β-actin* using the 2^(−ΔCT)^ method. Data are represented as mean ± SEM. Statistical significance is noted as **, *p* < 0.01.

We recently used tunicamycin treatment to characterize the UPR in the solitary alfalfa leafcutting bee [22]. To determine whether *Ldh* and insulin signaling components were altered in response to stress in the midgut of this distantly related solitary bee species, we measured the expression of this gene after tunicamycin treatment in alfalfa leafcutting bee females 7 days post-eclosion. We first confirmed that these genes can be induced in honey bees at this earlier age. Examining midguts of age-matched honey bees (7 days post-eclosion) after 30 μM tunicamycin treatment for 48 h, we found that *Ldh* and *Tribbles* were both upregulated in response to tunicamycin treatment (Figure 5A,B). When we measured the expression of these genes with and without tunicamycin exposure in alfalfa leafcutting bees, we again observed that *Ldh* and *Tribbles* were upregulated after tunicamycin treatment (Figure 5C,D).

## 4. Discussion

A critical first step in understanding the combinatorial impacts of disparate stressors affecting pollinator health involves defining common cellular processes that are impacted by multiple stressors, potentially underlying cellular dysfunction, tissue pathology, disease susceptibility, and mortality in honey bees [18]. This approach has been pursued to great effect in the fruit fly where it was found that heat shock, oxidative stress, and ionizing radiation all upregulated a core set of shared genes, although unique gene targets were associated with each stress trigger [48]. Prior utilization of this approach in our group revealed that genes encoding small heat shock proteins (SHSPs) of the *l(2)efl* family are part of a shared transcriptional response to proteotoxic stress [19]. These proteins have now been shown by multiple groups to be induced by abiotic stress [19,49,50,51,52,53] and biotic stress as they comprise part of the antiviral response in multiple bee species [54,55]. Here, we relied on transcriptome profiling (RNASeq) data sets we previously generated to identify novel genes induced by exposure to three proteostatic stressors in the honey bee [6,7,8] and identified the gene encoding LDH, also known as *ImpL3* in *D. melanogaster*, as one such factor. The expanded *Ldh* gene family found in Hymenoptera may be a functionally important feature of this phylogenetic group, but additional research will be necessary to better understand the role of the additional *Ldh*-like genes.

We observed increased levels of midgut lactate with increased *Ldh* gene transcription, suggesting that this metabolic change is connected with observed changes in gene regulation. Other change in genes involved in cellular respiration (*Sdha* and *CytC*) concomitant with metabolite alterations are also seen. Together, these data suggest transcriptional remodeling of cellular respiration at two points by diverse stressors in the honey bee midgut (Supplementary Figure S7).

Our observation of increases in some amino acids—including asparagine, aspartate, glutamate, and glutamine—but not others—alanine—broadly implicates an altered TCA cycle (as does decreased succinate described above). These metabolite changes may be a consequence of induction of the ISR by halofuginone in honey bees [7]. We did not detect gene expression changes associated with amino acid metabolism after halofuginone treatment in our previous work [7], but the NMR data suggest that amino acid levels are altered in response to this stress. Therefore, understanding the more specific alteration to the TCA cycle due to ISR activation or induced by other stressors will require further study. For example, AUROC analysis suggests that the aspartate/succinate ratio warrants further investigation (AUC = 0.990, MetaboAnalyst 5.0).

Currently, increased concentrations and activity of LDH is thought to impact cellular function by three mechanisms (Supplementary Figure S5). First, conversion of pyruvate to lactate by LDH reduces metabolic flow from glycolysis to the mitochondria, which may be important for mitochondrial health [56]. It is a striking finding that cellular stress caused by diverse triggers appears to modulate cellular respiration. However, it fits well with other data in honey bees showing that behavioral stress causes changes in oxidative phosphorylation and aerobic glycolysis [57,58,59,60], including changes in lactate and succinate similar to those observed here [57].

Second, by preventing full oxidation of glucose, LDH activity increases the production of various metabolic intermediates and contributes to the maintenance of optional cellular redox balance through an increase in NAD+ levels [61,62]. This has been demonstrated in both mammalian cell lines [63] and *D. melanogaster* embryos [64].

Finally, an important metabolic consequence of induced *Ldh* transcription may be increased levels of L-2-hydroxyglutarate (L-2HG). In both flies and mammals, LDH can use TCA intermediate α-ketoglutarate (2-oxoglutarate) to synthesize L-2HG [65,66]. In fly larvae, accumulation of L-2HG mediated by high *Ldh* expression is important for normal development [66]. Interestingly, however, increases in L-2HG levels are not observed in honey bee larvae [67], so this pathway may not be critical in this species.

Beyond the cell-autonomous effects of *Ldh* expression, increased lactate production can have impacts on other cells, both proximal and distal to the cells of origin. The lactate shuttle concept suggests that lactate produced in one cellular context can be used by other cells in a paracrine fashion (e.g., [68,69]) or even by other tissues in an endocrine fashion. Recent thinking suggests that lactate is continuously produced by cells in mammals, even under aerobic conditions, and can serve as a molecular link between glycolysis and the mitochondrial-dependent pathways, such as the TCA cycle and oxidative phosphorylation [70].

Lactate now appears to be a major circulating metabolite, at least in mammals [71], that may allow for endocrine metabolic coordination. A well-known example of this phenomenon is the Cori cycle in mammals, in which lactate produced by the muscles is used in gluconeogenesis in the liver [70]. A similar cycle has been proposed in honey bees [72] with some evidence in support. Additionally, it has recently been suggested that lactate may be playing an important role in the communication between cells and tissues beyond acting as an energy source [73]. In such a capacity, lactate may represent one link in the complex systems now known to orchestrate physiological responses in insects [74,75].

There are other potential mechanisms through which increased *Ldh* expression could be impacting honey bee biology. Organismal physiology can be heavily influenced by microbiota in insects [76]. This connection is especially important in honey bees with their stable coevolved intestinal microbiome [77]. It has been shown that bacteria within the characterized honey bee digestive tract microbiome can both utilize [78] and produce lactate [79], and lactate produced by overgrown microbiota in the fruit fly can have detrimental effects on digestive tract health [80]. It is therefore possible that the major effect of increased lactate is on the behavior or metabolic activities of bacterial cells within the midgut. In addition, our results do not rule out that bacteria of the microbiome are responsible for the increased lactate and other metabolite changes observed.

*β*-alanine is a known product of aspartate decarboxylation in bacteria [45,46,47]. Lysine and threonine, which are considered essential amino acids in honey bees [81], are also synthesized from aspartate by bacteria, such that the strongly increased aspartate levels in the halofuginone group support increased bacterial production of lysine and threonine in the midgut. Inhibited catabolism or altered transport of lysine and threonine into midgut cells from other tissues in the honey bee cannot be ruled out, but their correlation with elevated aspartate supports the connection to bacterial metabolism. Methionine, which is also synthesized by bacteria from aspartate, was not quantified in the NMR spectra due to limited sensitivity and confounding signals. However, it would be a good target for future study between stress-induced transcriptional responses in the host tissue and microbiome biology.

Our data suggest that *Ldh* is likely regulated by ATF4 and FOXO, perhaps acting in concert, which is in line with studies from other invertebrates. In flies, *Ldh* is known to be a target of ATF4 after ER stress [30,82,83] and mitochondrial stress [83]. Similarly, Nargund et al. [84] showed that the nematode ATF4 homolog, ATFS-1, leads to increased *Ldh* expression after mitochondrial stress.

FOXO has also been implicated in *Ldh* gene regulation in the fly [34,85]. The model that ATF4 and FOXO work in concert to upregulate *Ldh* and the other IIS target genes is supported by our previous work showing that genes encoding sHSPs of the *l(2)efl* family [19] and *Glycine-N-methyltransfrase* (*Gnmt*) [22] are part of a shared transcriptional response to proteotoxic stress. In other organisms, sHSP-encoding genes have been shown to be regulated by the same transcription factors we believe are involved in *Ldh* gene regulation, including ATF4 [83] and FOXO (references in [19]). Multiple lines of evidence also suggest that *Gnmt* levels are controlled by insulin in flies [86,87,88]. *Thor* (*4E-BP*) is also a canonical FOXO target gene in flies [89,90]. However, we did not see increased expression of the honey bee homolog of this gene (*LOC725039*) in any of the RNAseq data sets [6,7,8]. In *D. melanogaster* intestinal stem cells (ISCs), regulation of pyruvate metabolism, including via upregulation of *Ldh* expression, has been shown to be important in integrating cellular and organismal nutrient signals to control ISC proliferation [91]. Determining the relationship between stress and nutrient control in regulating *Ldh* in our system as well as the mechanisms through which such signals are conveyed and integrated will be important moving forward.

The potential regulation of *Ldh* and *SdhA* by FOXO and the upregulation of the IIS components we observe suggest that the IIS pathway is modulated by diverse stress responses in bees. The IIS pathway has been implicated in stress responses and stress resistance in *C. elegans* and *D. melanogaster* [92]. Some forms of stress in distal tissues in these organisms have been shown to regulate organismal responses through modulating insulin signaling [92]. For example, Owusu-Ansah [93] found that stress in muscles altered insulin signaling by upregulating the *ImpL2* gene, encoding an insulin-binding protein, also shown to be upregulated by divergent stresses in our study. *Impl2* has been shown to be very adept at modulating organism-wide insulin signaling in flies [39,40].

Intriguingly, there are also links between *Ldh,* ATF4, and the IIS pathway in lifespan and longevity. In *D. melanogaster*, *Ldh* expression increases with age and overexpression of *Ldh* reduces lifespan [94,95]. LDH activity and lactate concentrations have both been shown to increase with age in honey bees [72]. ATF4 mediates lifespan extension in nematodes [96] and one upstream activator of ATF4 regulates lifespan in the fruit fly [97]. The IIS pathway is a well-known modifier of lifespan in solitary model organisms such as the nematode and the fly [92]. Especially germane to this study, overexpression of *ImpL2* extends lifespan [98]. We do not provide evidence that *Ldh* or IIS components impact lifespan in bees. However, the effect of other factors controlling lifespan in model organisms such as dietary amino acids and NAD+/NADH ratio appears conserved in honey bees [99,100], making this possibility an important one to explore.

In model invertebrates, IIS activates juvenile hormone (JH) production which promotes *vitellogenin* (*Vg*) transcription in response to a high nutritional state, leading to pro-reproductive and pro-aging effects at the expense of maintenance (such as the preservation of stress responses) and survival effects. The physiological state of the organism switches to more pro-maintenance and pro-survival effects at the expense of reproduction in response to certain environmental stimuli, such as nutrient deprivation [101].

Sterile honey bee workers do not have a trade-off between reproduction and somatic maintenance. However, they do have age-based changes in physiology and behavior known as age polyethism that do appear in part regulated by the IIS [102]. Nurses are characterized by low insulin-like protein (ILP) levels and active FOXO-dependent transcription, while foragers have high insulin levels and express FOXO dependent genes at low levels [103]. Genetic or pharmacologic modulation of IIS function can impact this process [104,105,106]. In addition to developmental effects, IIS is influenced by behavior [107] and diet in this species [108]. Honey bees exposed to myriad environmental stressors are known to progress more rapidly through the stages of age polyethism, resulting in precocious foraging and reduced lifespan, both of which can contribute to colony failure [109]. Counterintuitively, the stressors we use appear to increase insulin target gene expression, which may provide a counteracting, but not fully effective, check on the detrimental effects of stress on honey bee lifespan previously reported.

Interestingly, we observe similar changes in *Ldh* and genes of the IIS pathway in both species of bees examined hereafter one model stressor (UPR induction), despite their stark differences in the degree of sociality. Honey bees live in eusocial colonies containing reproductive females, reproductive males, and tens of thousands of sterile females that perform all of the non-reproductive tasks required to maintain colony health. In contrast, female alfalfa leafcutting bees nest alone in small tubular cavities in stems or reeds in the wild, or human-constructed wooden or cardboard tunnels when in captivity. These females are engaged in both reproduction (mating and egg-laying) and completing myriad other tasks, including brood care (provisioning as well as nest construction, maintenance, and defense) and self-maintenance (nutrient acquisition, defense, and protection against stressors). Sociality thus appears not to impact either the transcriptional remodeling of cellular respiration or the insulin/insulin-like growth factor signaling after diverse stresses.

The ability to measure the relative stress of individual honey bees or even the colony through the use of biomarkers would be a useful diagnostic tool in the field. Based on the robust induction of *Ldh* transcription in response to a broad array of model stressors, quantification of LDH may provide an optimal biomarker for honey bee stress. Future studies to correlate LDH levels or activity with the presence of known environmental stresses such as infection or pesticide exposure will be important to confirm the usefulness of this strategy. Some evidence already exists that *Ldh* is increased in honey bees in response to many types of environmental stressors. For example, increased expression of *Ldh* in multiple tissues can be triggered by pathogen infection and parasitic feeding [110,111]. In one setting, behaviorally induced stress has also been associated with increased *Ldh* expression. Honey bees raised in ”high aggression” colonies, which are thought to be under stress, also show elevated *Ldh* in multiple tissues [112]. However, this is the first report of *Ldh* being induced by cellular stressors.

In addition to the importance of understanding how honey bees are affected by and respond to various stressors, there is great interest in understanding the factors that influence the health of non-*Apis* bee species [13]. This is especially true for solitary bee species, which account for over 90% of all bee species [113]. Our results suggest that *Ldh* expression levels may provide a potential biomarker of stress in some non-*Apis* bee species.

## Data Availability

All relevant data can be found within the article and its Supplementary Materials.

## Supplementary Materials

The following are available online at https://www.mdpi.com/article/10.3390/insects16030300/s1, Figure S1: Schematic of the structure of the four *Ldh* genes in the bee genomes; Figure S2: *Ldh* transcriptional changes with additional stressors; Figure S3: Honey bee metabolic promoter regions; Figure S4: Halofuginone induces ISR target genes and impacts survival in newly eclosed bees; Figure S5: Clustering analysis of metabolites; Figure S6: Correlation analysis of metabolites; Figure S7: Volcano analysis of metabolites; Figure S8: Schematic of potential cellular consequences of increased LDH activity; Figure S9: Schematic of observed transcriptional and metabolite changes; Table S1: Primer sequences developed for use in this study; Table S2: Study statistical analysis details; Table S3: Lactate dehydrogenase genes within selected non-bee insects representing various insect Orders; Table S4: LDH protein and gene IDs for bee species; Table S5: Lactate dehydrogenase genes in *Apis mellifera*; Table S6: Insulin/insulin-like growth factor signaling pathway negative regulator genes induced by stress in *Apis mellifera*; Table S7: All 30 aqueous metabolites measured and quantified by NMR in honey bee midguts with and without halofuginone treatment.
