# Supplementary material for: Remodeling of Cellular Respiration and Insulin Signaling Are Part of a Shared Stress Response in Divergent Bee Species
Source: Insects. 2025 Mar 13;16(3):300. doi: 10.3390/insects16030300 (PMC11942726; doi:10.3390/insects16030300)
Supplement: Supplementary file 1 [file insects-16-00300-s001.zip › Supplemental Figures.pdf]

**Supplemental Material**

**Figure S1. Schematic of the structure of the four *Ldh* genes in the bee genomes.** Honey bee genome (top) and the ALCB genome (bottom), with the stress-induced *Ldh* shaded in red (A). The predicted active sites (or homologous region) for the honey bee LDH proteins encoded by 411188 (193 – 199), 411187 (272 – 278), 411189 (201 – 208), and 725482 (214 – 221) and for the ALCB LDH protein encoded by 100881284 (190 – 196), 100877589 (201 – 207), 100881172 (202-208), and 100877481 (216-222) (B).

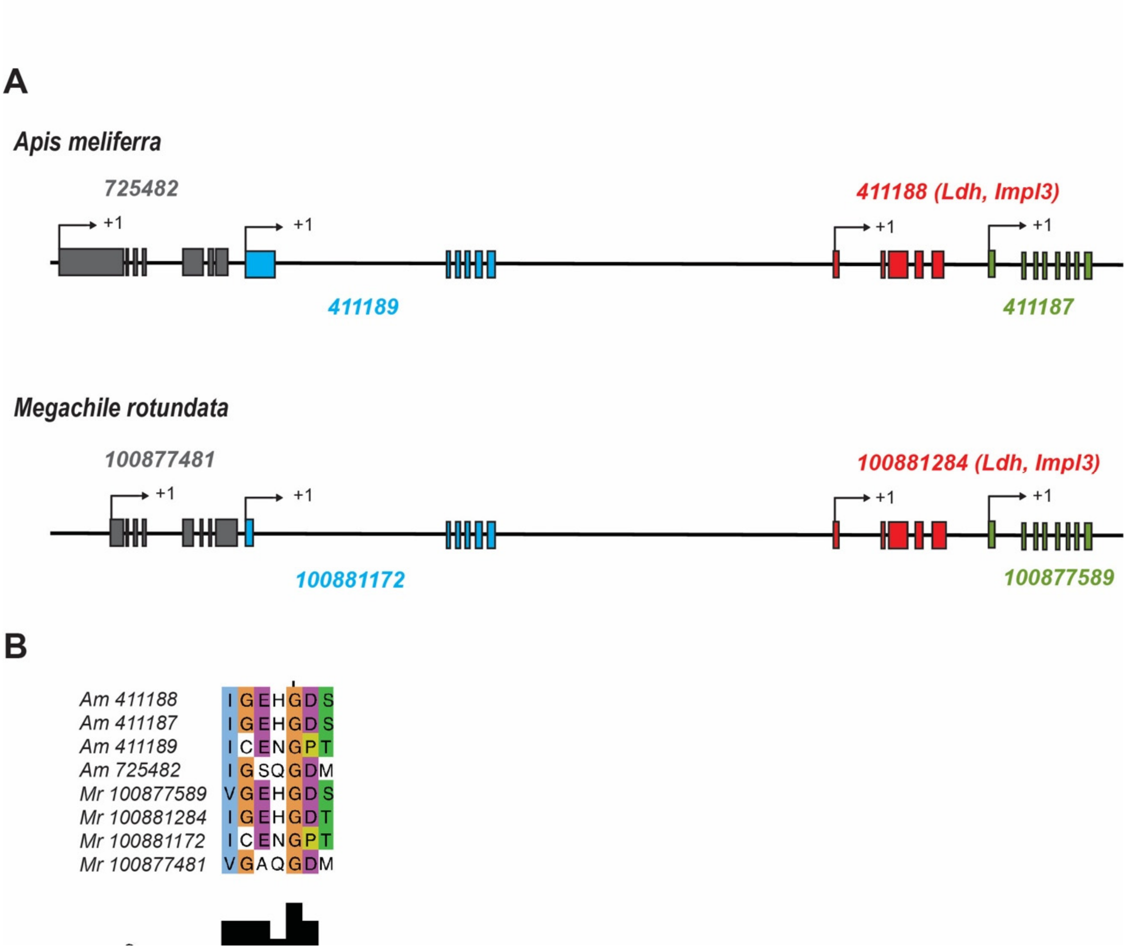

**Figure S2. Ldh transcriptional changes with additional stressors.** Transcript levels of the *Ldh (Impl3)* relative to *β-actin* in midgut tissue from adult bees captured at the landing board after pharmacological inhibition of tRNA synthetase activity (A), ribosome function (B) or induction of ER stress (C). Transcript levels of *Ldh* relative to *β-actin* in midgut tissue from queen bees or sterile attendant worker bees maintained for four hours in cages at either 35 or 45 °C (D). Mean ± SEM is shown and represents expression values of the genes of interest calculated using the $\Delta C_T$ method for individual bees. Statistical significance is noted as * $P < 0.05$, and ** $P < 0.01$ or a ≠ b where $P < 0.05$.

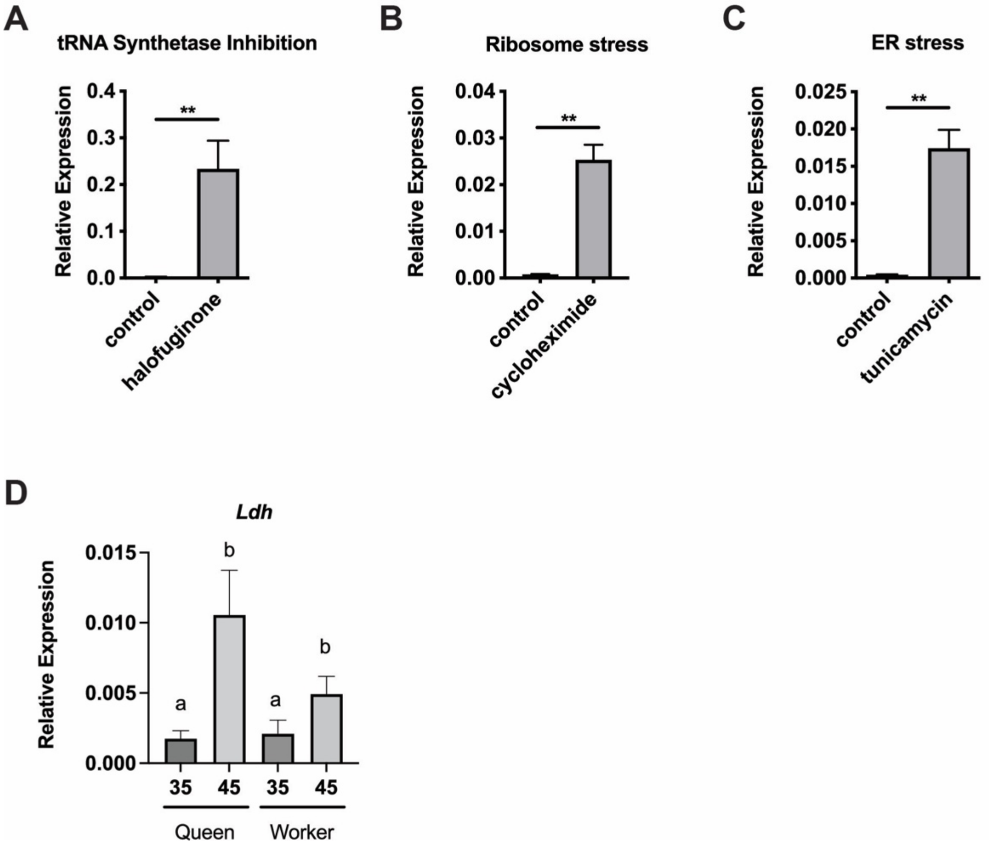

**Figure S3. Honey bee metabolic promoter regions.** Underlined region represents first 10 nucleotides of the transcriptional start site (TSS)

HSE (Heat Shock Element) consensus sequence = GAANNTTCNNGAA
FOXO binding = TKTTYACY
UPRE (Unfolded Protein Response Element = TGACGTGR
ATF4-binding motif = TTKCATCAK, TGACGT
AREs (for antioxidant response element) = TGAYNNNGC

***Ldh* gene (411188)**

GTTTGGCCATCGCTTGATACAAGGATTACGCAACTTATACGCCGTTTTGATGACATAACAGTT
ATGCATTGCCAATTTAACTTTCACACTGTAAAAAAAAAAAAAATAATAAAAATACCAAGCGCG
TGAAGCAAATTCAAAAGCTCGGCTTTTCTATTAAATTTCTCCTCGGTTTACTCGTCTCTACTCA
ACGAAATGCGCCAGCTATGGCGATATAAATGGTTGTAATTACGGGATGGCGGATCTCCGTTAT
TCAGAGAGCGTCAATTTCAATCCACGAGCACTTTTTTTTCCTTCCTCCCCTCCCTCTCCTCCTCC
TTTCAATGGCAAGCGTATAAAGGGCAATTGCAAAAACGCAGTGTATCTTTGTGTAATAACCTT
TCGTCCACTTCCGATTAAATTATCGATTAAAAATTCTTCGTGGAAGAAATTGAAGGTCTAAGA
TAAAAAAACTGGTTAATATAAACAAACTGTAGAAAACGATATATTACACATTTTATACTCTTT
CAAGGATATAAGTAAACTTCCTCTTACTAGGGTTTTTCTTTTATCGTAAAAGTAAATTTTAATT
CGTGTTTATATAAAAGTATATATATATATATACGTGTACGTGGTTGATGAGAGAACAATATGC
GTGTCATCATTTTTAAAAAGATATGTATTTAAAAATTGCATCCAATTCAAACAATGACTCGTG
CCTCGCGTTTTCTCGTCGATTATCATACGTGTGTTAGATTTACGCGAGGAGAAAAAGGAGAAT
GATCGCGAGATGAGGAAACGAGTAGGATGATGAAAAGGTGTCTGTGATCACGTAGAGCCATA
GTTTCGTTGCGTCATTCTCGAATGCATTTGTCGCGGTATTCCTGAATTATGATTCAGCCGAAAT
ATCATTCGTTACCGAGAAGTTGACGCCGTCGATAGCGAGTTTTGCTATCGACGAGAGATTATT
GCGTCAGATAATATTGGACAAAATCTTGATATAATTGAATAATATAATTGAATTTTTTTTTTAT
TAGTCAATGTATATTTTCTTCAATTCGTTCATCATAACTATATATATATATATATAATTTATAAACT
TTCTATTGTTTGTTGAAGAAAGAAATATATCTTTTCTTTTTTTTTTTTTATCATATATCGAATGT
ATCGAATATAAACACATATTGGAGGAGGAGAGAAAAAAAATGAAAGTACGAAACAAAAGCT
AAATGCGTAAAATGTCGTTGCAAGTTTGCAACGATAATTCGGTTCCTCTTTTTCTTGGTAGCAC
ACAAACGTGTGCATCGCATCATTGGAATATAAATCTGAATCATATAACGTTGTTCACGCGTCT
GTAAAAATTTAAAAGTCGCTCGTCCGAATTGGACCTAATCTCGCGGATGAATTGTTGAAAATA
TCGATCGACAATTTTTCTCGGTTTTTTCGGAAGTGTTGCTTCGCTGTAAAGGTTATCCTTTATTA
CGATTGAGTAATGGTCATTTAATTCAACTGGTATGTAAATTCCACAATTCCGATTAAGACTCGT
ATCATAATCGTTTCGAAAATGATATCTTTGCGTTATCCGTTTGACGGACAATGATAAAGAGTG
AAAAAAAAAATTAGAAAATTTCTGTTGCATGAATCACAGATGACAGTGAGAATAAATTAAAC
TAATTAATTCGTTAGATATCGCGATCCATGTATATGTACAAAAATATAGAATATAAATCTTCG
TATACAAGAGTTTTCTCCTATTCGAATAAAAAAGGGAAAAAAATTAAAAAAAAAAAAAAAA
AAAAGGGAAATAATTAATTTCCCTTTTCAAAATGTTACAAAACTCGATTGAGCAAGATAGGAT
AAATTTAACGGGATCGAAATAAAATGATCGTATCCAGCGTTTAGCTAGCATTCCACCGAACCC
CCAAATGTATCGTAGAATGTATTTTTAGCCTCGACTGAAAACGAACGAGACTCATCCTGCGGG
CTGATTTTTGTTGACAGGCACAGTGGGGATCGAAAAGAAAGTACAGGACAGATCGATAAACT
TATTTCACGGAAGGGAACGCACGTTGCGTCATTTTACGCGTGCGCAACGATCGTAGCTCGTAG
TTTCGAAAATGTATGCCACGACGTACGTGCTCCGCCGTGCATCACCACCCAGGTTATGTATTA
CCGATTACGGATTTGAAATAACGTCGATGCGCCATGTGTACAAGTATTATCGACACGTGATAT
GGGGCCACGGTGAAAATTTGTCGGAAAACCGGACGCAAATTTTATCGTTCCGGCAATACTTGG
CCAAATCCCCGATTTTGCCAGACCCCAATACGCCGGCCGAAGTCTATGGGAGGTATAAAAAG
CGGGGACAAGG**TCACCTTAGC**AAACAGAAGGCTGGGAGCAGTCGTACGGAACGGAAGAAAG
AACGGCTGGTAAACCGTCCAAATAAATTTGGTGTTTGCTTCATTCTCTTTCTTTGCTTTGTATCT
TTCTTAAAAGCAATCATGAGGTTAGTGTACACACGTTTATTTCTTTTTTATTTATTTATTTATTT
ATTTTATTTTATTTTATTTTTTTTTTTTTTTTTTCATTACAATCACGTCGCGAATTTCCAATTATA
CATTCGACAGAATTGTACTTCATCGATCGACGATCGGTTCGAAACGACAAGTGTGCATAAACG

CGACGAAACGTGTAAGGACGAAACCGTGTTAAGCTATGTGGAATACCCATAGAAGAAACTAG
GTCAAGATGCAATTACGAGAATTATTCGATCCGATGGTGTTCATTTAAGAAATTTTAACCTCA
TCTTGCTAGTAATGTTTCCTTTCGGTGATGGATTCTATTCAAACCTTTTCGCTCTGCTCTTTCTT
TCGCAATTTGAATTTGAATTCGATTTGTTCGAATAGTTGCTTTGTACGTAACCTGCGCTCTCGT
AGCTTATCTATCGAACACCGGTTAACGTACTGCTAGTGGCATCTTACGAGCTTGCGTATTACTA
TATTGCTATACTATTAACGTAGTACGAGTGTAGTACAATCAATATAATTTACAAACCAGCGA
AATTTTTGCTTCTTAATTTTTCGTTGAATCGAAAAATAGTTGCAAAAAAATTTCATTTCTGGTT
TGTTTAATGTAACAGTAGATAGTAATGTTTTTTTTTCTTTCTATCTTTTCACTTTACGATCAAT

***SdhAl* gene (*408734)***

TATCGATCTTCTTGTTCATCGAATGCTTCTCAGGAATAGGAAATCAGAAGAATCGAACGCATA
TATTTGTAATGTAAGCATGAAGCGAGAGAAAGAGAGAGATGGCAAATTTCTTCGACGAATTTT
CGTTTTATCGTCTTCCGTTTCCTCTTCCATCGCGGCCACTTTTACCACATTTACGTTCTTTACTT
CGAGCATCTTCTCAAATATGCCTTTTAATGTATTTATTATTTATTGTTTTTTTTTTAAACGAGAC
TTTTACATCACACACATACATACACATTCATATACACATATACATACACAATGCACCGATGTT
CGAGCAAAAGAAAGAAAAGACGTTTCTTTTCGAGATTTTTTCCTATTTCGAATTATTAAAAAG
AAAAAAATATCGATCCATCGATTGTGGAAAAAAAAAATATCTCCTGTAAAGAAACGTCTCGTT
GAATAGAAGGAGGGATGAAATTTTGAATCTTTTAATCGTGATTTTTCAGCGGGAAGGATTATG
ATCCTTTCTTATTGGCGGAGCAACAGATGAACGACCTGCTCTCAGACACCAGCGAGCAATCCG
TGGCGGATAGCTGTCCATCGATCGATCAAAATTCCCGCCAAAAATCATTCCCTCTCTCTCATTC
CTCGGCATTCGTCAAATATCCTTGCCAATCCTCGCCCAACGCCGGCCACGAGAAACGTTCCTC
CTCGATACCAGCTCCCCCAACCGAACTCGACGACATCACCTCGGACTTCTCGAGCGATTCCAC
CGAGACGAACTCGTTGTCCCGTGAACTTTTCTCTCTGAAAGACGAGAAAGAACGGGCGAAAA
ATTGTAGAGGGACGAATCCAGAGAGGAGATCCCCGGTCAAGGAATTGGGTAGACGGGTGATC
ATAGATAAATCGAGAGCGTTGGGAGGGGACGAGTTGGTCGAGGAGGGGAATAAAAGCGTGG
CGGGCGAGGCTCGTAAGATCGCGGAGCCTAGAGCGTCGGTAAAGATGATTCGTCCTGTTGTCG
ACCGTTCCCCGTCCGTTCGCGCCTCTTCCGCGCCCAAGGCTGGCCAAGAACGCGGGAAAAATC
CCGCGTCGAAAATCCATTCCCACGACGAGAACGCGTTGCCGTCACGTAAAAATAAGAATTCG
AACGGGAGGAATAACAATTATCCTTTGAATCTTTCGAGCAGCAACTTGAGCCTTAGCTCGATA
ATATCCTCGGACGTGGACATGAAACGATCGAATTCAGTGTTCGACGAGTTGATGACCTCGTTC
GAGGAGGACGAGAACGGTGCGTTCGTTCCATCCTTGAAATCCCTTCTGAAAACCGATTCCTTG
TCCAGCCCTGTCCACGCGTCCAGACATCGCAACGGCCGTATCAGCGACGAGGAACTGTCTTCG
CCCGAGAGTTACAAGAGGCAGGATCACAATAAAATGAGCGGAGATTCCGCTTACAGCAGGTA
AAAATTCGATGTTATTCGGATATGCCTTCTCTATACTTATTTTCTGTCATTCGAATTCGAAGCA
CAGTGTGTTATGCTCCCGCGTTACGCACCTAAGAATTACCCTTCCATTTCTTCCTCCTCTTTAGT
TTAAATCGCAAATATTCACACCACGGACGCAGCACCAACGACGTTGCCGGACGTTTCGACGA
GGATACGTCGAGGAGCAACCGTCGCGACGGGGACGGTGGTGGGGGGATCCACGTGGTCAAGT
GCAAGATGTCCAAGTATTGCCATCAGTGCGGCTTCAAATTCCCCGAAACCGCCAAATTTTGTT
GCGAATGCGGCATCAGGAGGCTCGTGCTCTGAAACTTTCGGCTGGTCGACAGCCATGAACGA
CTTCGAAGAAGAAGCGATCAACTTCGAGTAAATTGTCAAACGATCTTCGATCGTAGACATTTT
CTTTTTTTTTTTTCTTCTTTTTCTT**CTTCTTTGAC**AAAAAAAGGCAAAAAAACTTCAAAACTTT
AAAATTGCAGATTAGATGATAGAAAGAACGATTCGAATGTAAACACAAGGTTCAAGAACAAA
TGCAAATGTCGAAGCACGGATGATGATAAAAAAAAAAAAAATATATATATCAGCACACGAAGG
AATTGAAAAGAAGAGTTTATTTTTAAATATTCGTGGAACTGTTTAGAGAAGAGAAGAATTTTA
TTCTTGCAGTTTAGAATTGAAAGCTATGTATCTATCTACGTATACTCTAGTCACACGTTTTGGT
CTATTGAAATTTAAAGGAAGAAGTTTTAAAAAGAAAAGAAACGGTGAAATTTATCTGGTATA
TCAGGGAACACGGAAGAGGAAGCAGAAAGCAGTCAAGCAAGAGATAGCTGAGATTTCTATTA
TATAATATATATATATATATATATTTTGATTAGAAAGGGAGAGAATTAGATTTTAAAAATTTAAT
TGATTCAAGATTTCACGTCCTCCCAATATTCAAGGATCAAACTTGTTCTATGTGAAGAT

***Impl2 gene (409512)***

ACGATAAAAATCCGAATTGGCCAGTCTGTCAATAAACCGTGGTTAATTAAAACTTCTTCTAGT
TATAAATATTATTTCTTCTTCTTCATTTTTTTATACGTACTTTTTTTCAAATAAAAAGGAGAAAT
CCTTTCTATTCGAAATTTCATTTTTTTTTTTTTTTTAATTCTTAAAAATGATACATATATTTCTATA
TATAGATATCATGAACATAAGAAACAGATAAAAGAACAAGAAATAGATAAAAGAATTTTTAA
TAATTTATAGTTTGAAGAAAGAAAGAAAAAAAATTTTTCTGTGTGTTTGATTTAAAAATATGT
TATATTCTTCTTTCAGAAAAAGAAGAAAAAGAAGTAAAATAAAGGAAAAAAATATACGAGAA
AGAAATATTTCTTTCCTTCTTTTTTATCATTTTTTTTAATAATATAAATATCTCATATACATTTA
CAAGAAAGAATTTCTTTTTCTTTTCTTTTTTTTTTTTTTTTTTTTACTTTTTCAAAAAGAATAAA
TTAATATTATATTCTTGCTTGCATACATCTAGAAATAAGAGGAGAGTAAAAAATTTTCATTCAT
ATTTCATCTTTCGTGTTTCATACTGCATATTTCCTTATCGCGGTTTACGGTCTCCCTGATATTAT
CTCTAGATTACGATGCGTTAAAGGGCTCCGCGGAAAGAGGGCGCGAGATCATAGGGAAGATG
CGAAGATTGTATAAGAAGTTGCATCATTCGGTTCGAAACCTCCTCCCGTGATGTGCGCTCCTA
GAAAGCATCTCCGGCGCGAGTATCTTTATCGAAAGTTCTATCTCGTAGCAGTACCAGATTCGA
TATTTTTGCGGACGTTTTCTACGTGTGACAATTACCCATCGTGATTTACGAGTTCAAAGGTAAG
GATAGATTCAAATTTTCAGTATTAGTATTAACGCCCCGCTATTAATATTAAAATGCTACGTGAC
CACGTGCAAATAGATTTTTACGTGGAAAGAGAGATAATAGAGATGGTGAGGTGAGATTTGTT
AAATAGCAGAGATAATCGTAAAAGAAATGACAAGGCGGACGAAATTTTAGATATATATAAAT
ATAGATTTTTGAGAAGTTTATATTACTTCTTAGTTTCTCGAAAGCAGATAACGTACGGAGAAC
TGCATTCTATTTTTAGTTACATATATTTTAACAAGCAAAATTTCGACCAACTTTCCTCATTTTCA
CAGGCTGTCCGATTTCTCTTATTTTCTAATCAGTTGACGCTTTTCGATTAATTTCATCATAATGT
TAGAGGTTTAGTGGTTTAGAGGTGTTTAAACATTGCTTCGCGATAACATCATCTCACCTGGTCT
CATATCAGTTTTTTCGATTACATTCCAGCTGTGTCGTAATTTTTAACCGCAGAATTATACTAAC
GCAATATTATTAATCCTTTCGATTGCTCGGTGAAAATTGAATTAAATTATAATAATCCCTCGAT
CTTAAATAATTATTTTATTTTAGAATATAAATTATTACTATTTTTTATGCATTTATCAATTATCA
AGATATAAAAAAAATTTACAATATAAAATTTATAATACAAAATTTATATCCGGAATATTTAAT
TATATATTCAATAATTAACGTAATTAAATTATCAGATGGTATTCAATTTCTAATTTTTCAAAAG
AATGTGCAAGCTACTAAACACATTTAAGTAAAACGTGCTTAAAGAATATTTCCCCCGCACATT
TAAAATATATTTACATTCGTTAAATGTTTCGGATAACATTGTTCACATTAATACCTTGCCGCTT
AGATTTATATAATACTTTTTGCTTGCGCAACTAATTATTTTCCAGATAAATTAAACGAAAACAC
CACCGTGCCATTTATTTGTAATTTAAACACCACTACGATCTGTAATAATCGAAATCGAAATAT
CGAAGGAAGTTTACTTATTCGTCTCACTTGCGAGACGATAAACCGTCCAATATCTCGGAAGTT
TTAGCCACTTTTAACTCCTCCTCGGGGACACGCTGCATGCAAGTGTTTGCAGCAAAAAAAAGT
AACGCACACCAACACGTCGCATCGTGTATCGCCTCCAGATTCTCCCTTATTTTTTTGCACCGCA
TGTGTGTTCATTAACACGCTCCGCGCGCGAATTCATTTCCAAACATCTTGTCTCGTTAACAAGC
CCAAGTTACTTAATCTCCTTTAAACGGTACGCGTGACCTTCCTTCCTCCTCTCGCTCAGTAACG
CTCGTCAGTAACGACCGTCACGGGAAGGAAAAGGGGGAGCGAGAGAAAGCCGAGACGAGAA
GACGCC**AAAGATAAAAT**GATGCGGTAGCATCTAAATTTTTATTCGTGAACTTGGTTGAGAAG
GACCGATCCCGAAAGGCGAGGGAACGCCTCAAAGTCACGGTAAACGACGCTGCCAGCACGTT
CACGCATTCTAAAATCTTAAACGGACGTGTCGAAGCGGAGGAACCGTGAGAGAGACACACTG
ATTTAAGTCGAAGAGAGAGATATATGTCATTCGGGTATTCCTCGCGCGACACGTTGCCTGTAT
ATCGTCGGAAGGATACAGGGCGAAGGTATCGAGTTTCGTGTCTTGTACGAAGAGAATAAAAT
ACGATAGAATAAAGGAAATCTCTTTAATTTGGTGATTTCGATGCTCACGTTAACGAGC
AGGCCATGCGACAGATTCAAACGCACGACTTATGGAATATAATCCCACCTGAGATCGTTTCGA
GATTCTCGTTGTGTGAAGAAACTCGATTCTCGTATCGATTTCTTCAGTTTCGAAATTAATTATC
ATTTCCAAATAATTTTAAAAAAAAATCTTTCCGTGATTCTAGTTAACAGTGACATTGAACGAT
CCCTGTAAAACGTGTCGAGT

***Trbl gene (409288)***

ATGGTTAATTCTCCCAATTTGTTAAATGCTGCTCCGGCTGCGGTGAAAATTTCACCAACCTAAA
ACAATTAATATTTGAAATGCTTGAATGATTTGTCGTACAATTAATATTTATATTCTCTTCTTCTC
ACAGACCTTCATTAAATAATTATAAAAAGAGAATTTATATCAATTTATTAAACAATTTTGTCG
AAGAATAAAGAAAAAAAAATATTAAAGGAAATCATAACCTAACCTTATGCCCGCTTACAGAC
TCCATTGCTAGAGTACTCGAGGGTTAAAGCACGTGACTCACGTAGAAAAAGGAAGACGAGCG
GTGGGGGAGAACGACCGTTAAGTGTTATATGGCGGGAAACTGCCTAAAATCGGCGACAACAG
ATGCCTTTTAGCTTACCTTGCTCGCAGAGTTCATGTCAGTTTGTTGTTATGCTCGTACTTCGCCA
TCAGCAGAACAAACATTTGTACGGTATTGCCATCTCGCGTCGTTCTCAGAATCTTTCCCTTCAA
GTAATTGTAACCTCTACTAATCTTTATTATTTACTCTTTTTCCATTTTCTCTCATTATTACGTCAC
TTTATATAAGATTAATAATAATGGACATTTTTATCTTTGGTTAGTTAGAAAATTCATATCGACT
TTTTATATAATATATGAAAAAAATTTATACGAGTCTAAATTAATATTTGGAACATCTATATATG
AATCACGTACAGAAATATTTCAATGTTTTCCAATTATTTCTGACGTTCTAAAAGAAGAAGTTA
ACGCAAGAATAAGATAAATTTATACGTATTTTTATATTACAGATAAAGAATAAAGAATCTGAC
GTACAATTTGATATTTCAAAACGATTATTATTATTATTTTTCTTATATTTGAATATTCAATTTCA
GAAAATTAAAATTATTTAAAAAAAAAGACAAAATTATTTAAAATACAATAGTTAAATATTAATG
TATTATTAAATTAATTGTATTAAATCAACGTATCAAAATGAAACTTGAAGTTAGTCAATTTTAA
ACTTCAACATATTAATTAATAACCTTACGGAAAATTAAATGTATAAAAAGTAAACAAAAATTT
CCAAAAATTTGGTAACCTTTCTTTGATATTTTTCATTAATGCAAGTCATCTATCTTTTTTCTTCT
ATGCAAATCATCTATCAGAAATTATTCCGCCAATTGTATTAATATTCTTAACCACATTCGCATA
AAATATATATATATATGTACTATATCCTCGTCGCTGGTTCGACGTCGATTAGCGTACGGAAAT
GCATCAATCTGAAGAGCGATAGCTCACGTCCTCGCATAAATTAATAGTCTCCGTTTCCACTCA
TTGTTCTTCGTAACCATGACTCTTCGAAAATTACAAAATTCCCTTAATATACGTTCAATATGTT
CAACAGGATCTCCGGTGTATTTCAACGTTTTCAACAATGGGAAATCCTCGGCCTTCGAACGTC
TAAGCCTTCTGGACGCGTTTCTGTAACATTTCCAGACTTGCGGCTCGATCACTATCATTTCGGC
TACGGAACAAGCCTTTCTCAGAAATTTCACCAGCCCTTCGTCCCCGTGGTTCAAGTGGATCCA
CATCGTAACCGAGAAACAGAACACGACGTCGAATCGTGACTTTTCGAATCGGGCAAGATGCT
CGTTCAGGAATCGGTTACAATCCTCGGTCAAAAAATCGAGGCACTCGAAAATAATACGATCC
GGGCGTGGATTGCGTTCCCGTGCCCGCTCTATTAAGATCGGATCGAGGTCCACGCCGAGCAAA
CTGATCTCCTTCGACTGATCGGCCGATAAAGCTTTCTCGAGGAAGTCGTGCAACACGAATGTT
AAATCCTGAAATCGTTAAATACGATTTTGCGATATTTATACGAGATACCCTTTGATCAATGAA
TTTTTTTTTTCTAAATATATAGGATCGAAAGAGAAGAATTTTTTAAAATGCTTATAATTTTTTTT
AAGTTGAAAAATATTTTTCTCATAAGTGGAAAAGAATTTCCGGAAGGAAGAACGATCGTCGA
GAGGGGATAAGATCGGGCTAAATTTAGACGGTTGTCGTTTGTAATTTTCGGATCACGGTCACG
ACTCATCCTATTTTGTCTGACAGCTGTTTCAGCCTTACCCCCGCGTTACAGCCGACGTCTAGGC
CTACGTATTTCCGGTCCGGATGGGCAGACCGCCACACGCCATGCGGAAGTTGTCGCACGCGCT
CTTCCGCAGGATGGAATTGGTAATAATTCAT**GAAGTTCCCG**TGTCTACTAGCACCCGGATCCG
TCTTATCTTCCTGCCTTTCCTTGCGCGATGATCGTGCCTCTTCCCCGGACATTTTTAGAAAATCT
GAAATTCGGTGAGTCACATTTTACGATTAAACCTCGTAATATATTTTGAACGTTTAAAAAAAA
AAATATATCTTTGAACATAATGTAATATAAAACAAAATCATAGAAATATCTCTTGATTAAAGA
TTATATGCAATTTGAAAGTCAATTTCCAATGCCTGTCACGCAAAATATCTCGAACTTTCATAAA
TATTTCTAAATATAATAATATGAATTTTCGATAGAAGCAAGTAAAGAATTTTTTATAGAAACA
CGGTTAAGATATATCGAGTCTATTAAAATCCCTGTGCAATGCAACAATAGACCGATCTCTCAT
TGGAACTTCATCACCCTTGCAATTATATAAATCGGAAAGATGCTCGGCCTACTCGCGATTTCG
TAACGCCGTCGAGACGGCTGTTTCGCGAAAGTTCGCGAGTTAATCGGTTAAAAATAATGC

**Figure S4. Halofuginone induces ISR target genes and impacts survival in newly eclosed bees.** Survival of individual newly eclosed bees fed sucrose solution containing 200 µM halofuginone (n=92) or Dmso vehicle (n=97) starting on day 4 post-eclosion (A). Transcript levels of the *Ldh (Impl3)* and l*(2)efl 410087a* relative to *β-actin* in midgut tissue from newly eclosed bees feeding of sucrose solution containing halofuginone (n=8) or vehicle Dmso (n=8) for 4 days (B). Mean ± SEM is shown and represents expression values of the genes of interest calculated using the $\Delta C_T$ method for individual bees. Statistical significance is noted as * $P < 0.05$, and ** $P < 0.01$.

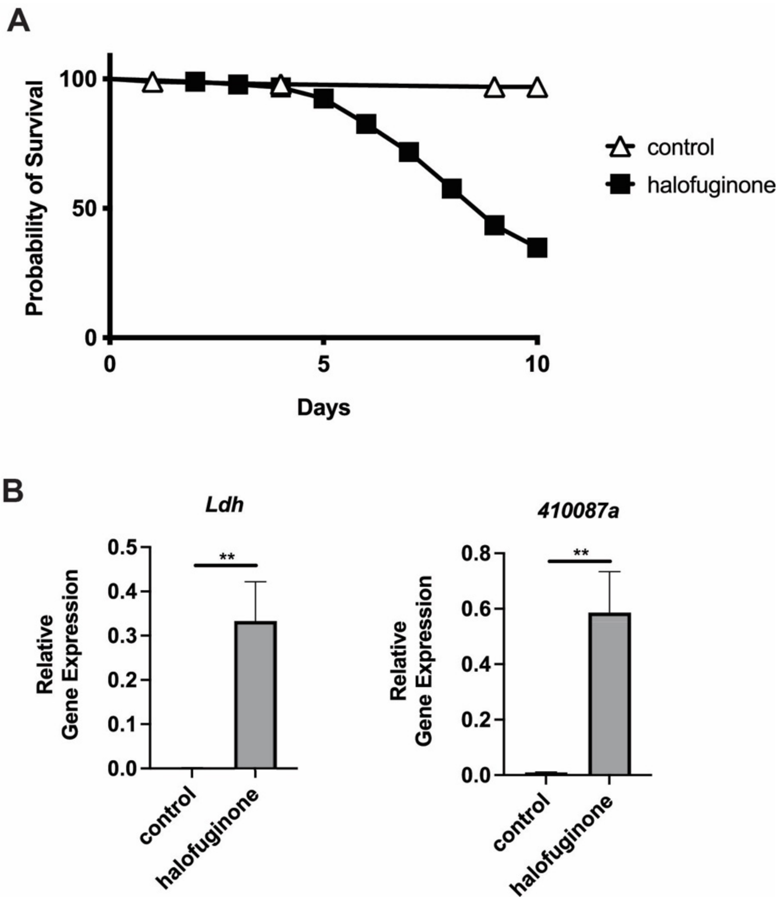

**Figure S5. Clustering Analysis of Metabolites**. Supervised clustering was performed with (a) PLSDA, (b) sparse PLSDA, and (c) orthogonal PLSDA models (MetaboAnalyst 5.0), shown with associated variable importance scores (VIP >1.2). All models achieve separation of the control and halofuginone groups; the sparse PLSDA model was constrained to 5 variables. Note that univariate ROC analysis of aspartate alone results in AUC=0.89. Log and Pareto scaling was applied, however similar results are obtained with different scaling strategies.

(a) PLSDA

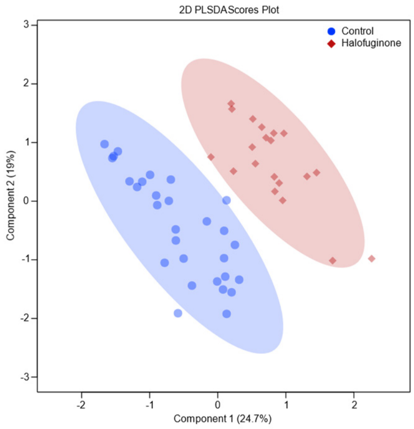


| Metabolite | VIP (C1) |
|---|---|
| Aspartate | 2.39 |
| Glutamate | 1.80 |
| Asparagine | 1.78 |
| Lysine | 1.57 |
| Succinate | 1.45 |
| Sarcosine | 1.37 |
| Threonine | 1.26 |

(b) Sparse PLSDA (5 variables)

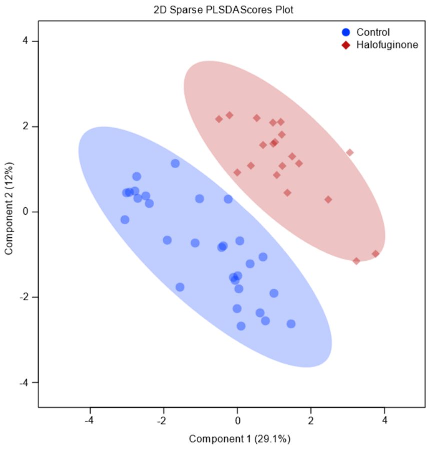


| Metabolite | VIP (C1) |
|---|---|
| Aspartate | 0.750 |
| Glutamate | 0.497 |
| Asparagine | 0.394 |
| Sarcosine | 0.175 |
| Lysine | 0.065 |

(c) Orthogonal PLSDA

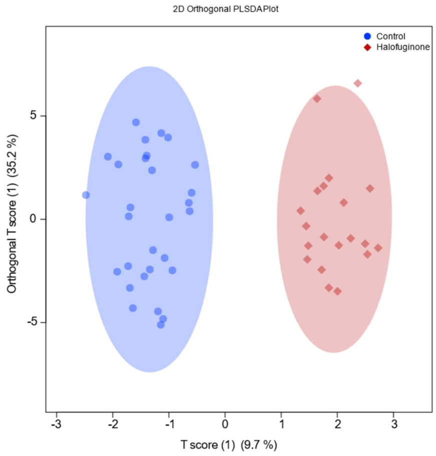


| Metabolite | VIP (T1) |
|---|---|
| Aspartate | 1.98 |
| Glutamate | 1.85 |
| Asparagine | 1.75 |
| Sarcosine | 1.62 |
| Lysine | 1.35 |
| Succinate | 1.34 |
| $\beta$-alanine | 1.30 |
| Glutamine | 1.28 |
| Lactate | 1.24 |
| Threonine | 1.24 |

**Figure S6. Correlation Analysis of Metabolites.** Correlation plot (MetaboAnalyst 5.0) of the targeted metabolites considered in this work support trends noted in other analyses. Note for example the strong correlation of aspartate and glutamate (Pearson coefficient = 0.815, $P < 0.001$; see also Figure 4 of the main body), threonine and asparagine (Pearson coefficient = 0.831, $P < 0.001$), and glutamine and asparagine (Pearson coefficient = 0.737, $P < 0.001$).

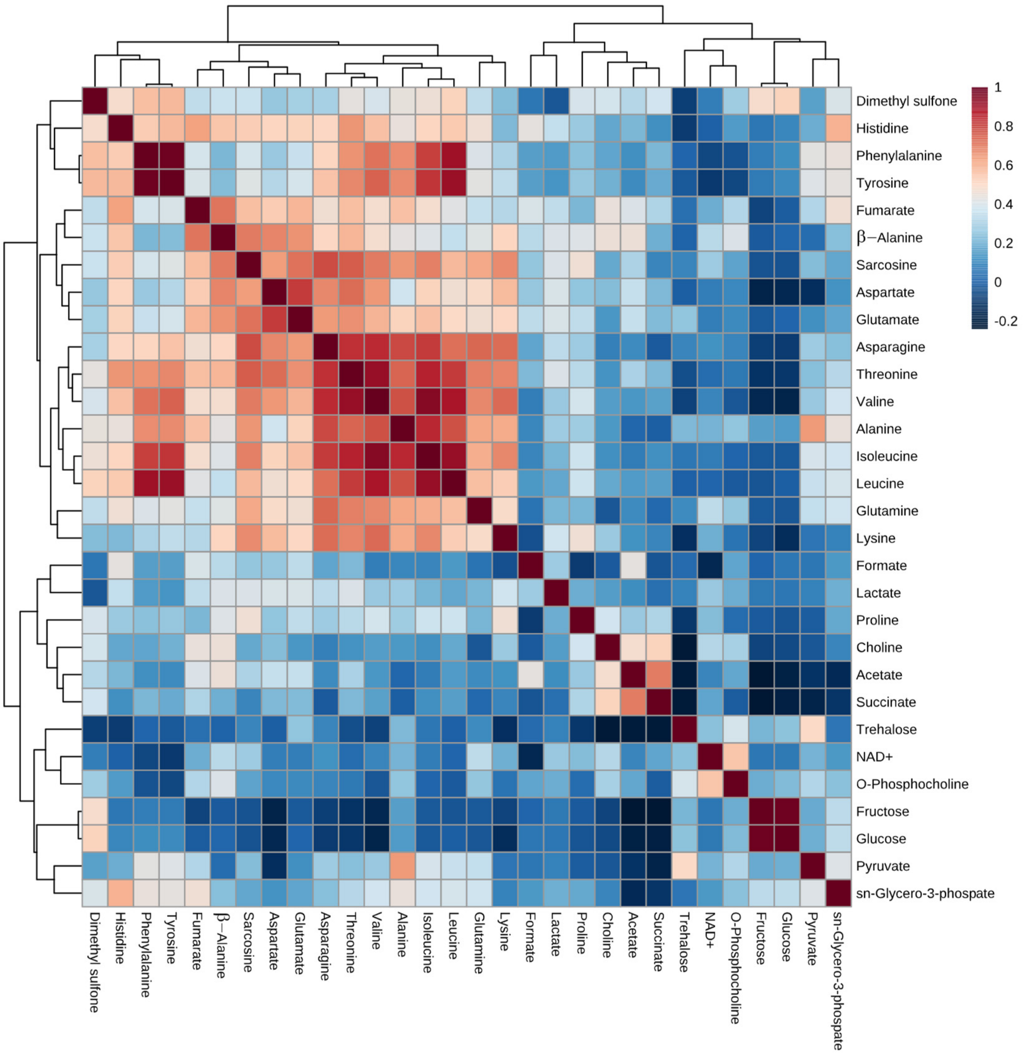

**Figure S7. Volcano Analysis of Metabolites.** The volcano plot is shown that employed for simplicity solely nonparametric comparisons (MetaboAnalyst 5.0) of the targeted metabolites considered in this work.  Overall trends are unchanged relative to Table 1 of the main body.

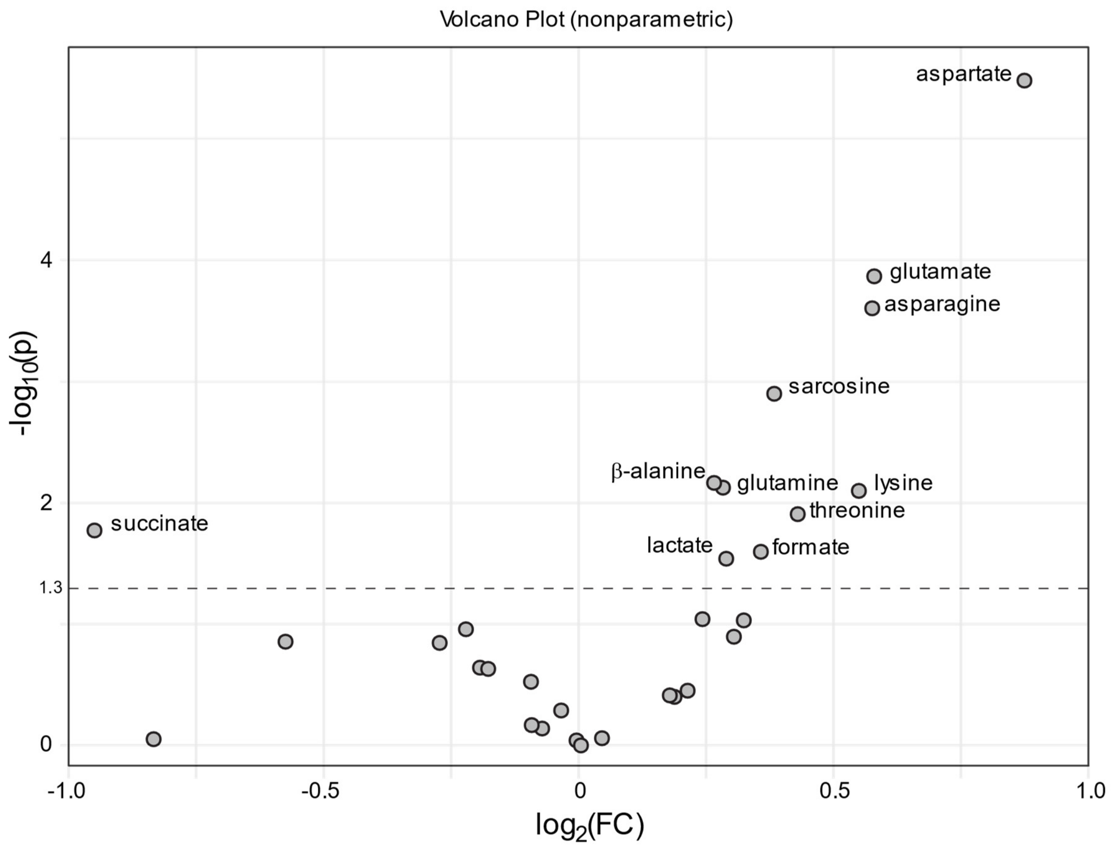

**Figure S8. Schematic of potential cellular consequences of increased LDH activity.** LDH mechanisms via reduction of pyruvate flow through cellular respiration and NAD+ regeneration (A) and via production of L-2-hydroxyglutarate (B).

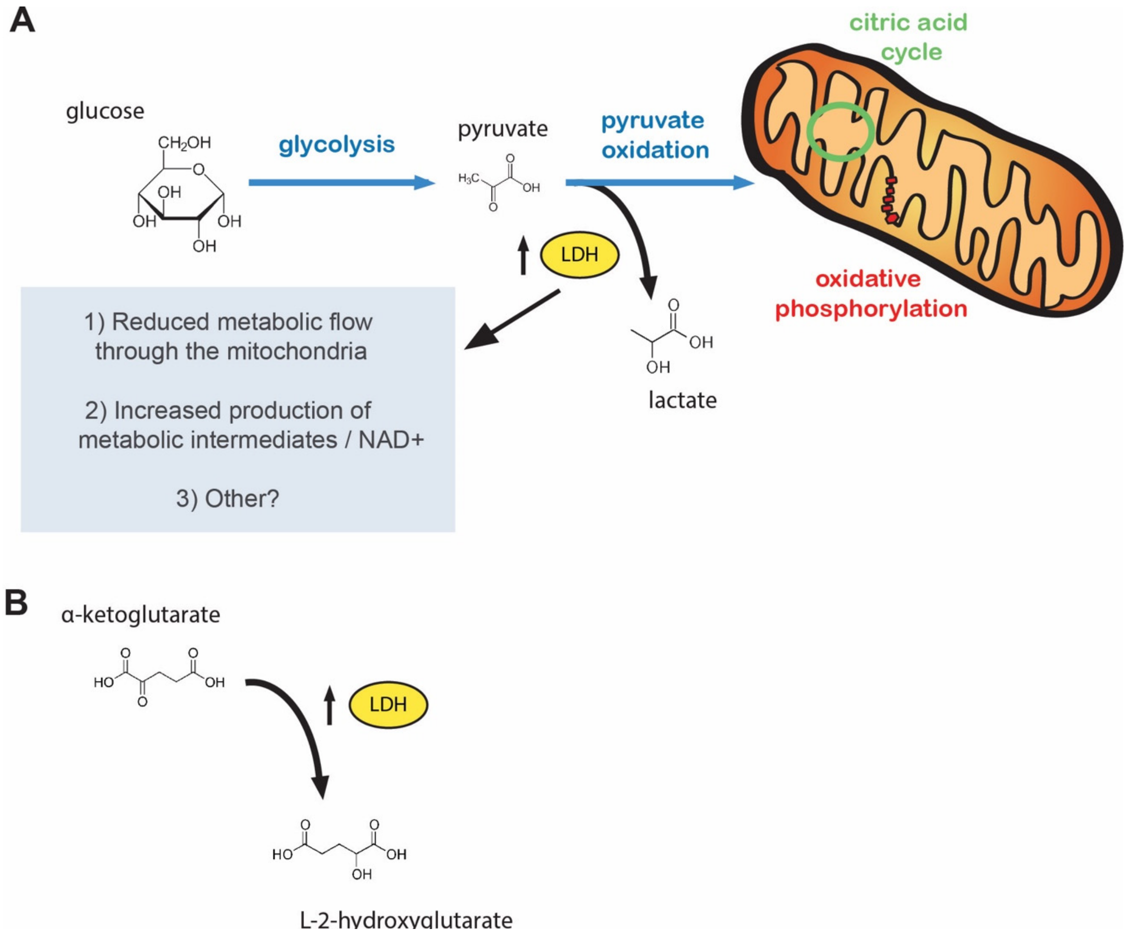

**Figure S9. Schematic of observed transcriptional and metabolite changes.** Transcriptional (bold italics) and metabolite (bold regular) changes (green = increase, red = decrease, black = no change) associated with cellular respiration. *Lysine and threonine are considered essential amino acids in bees (Groot, 1952) so increased levels must be due to changes in cells of the microbiome or transport into the midgut from other bee tissues.

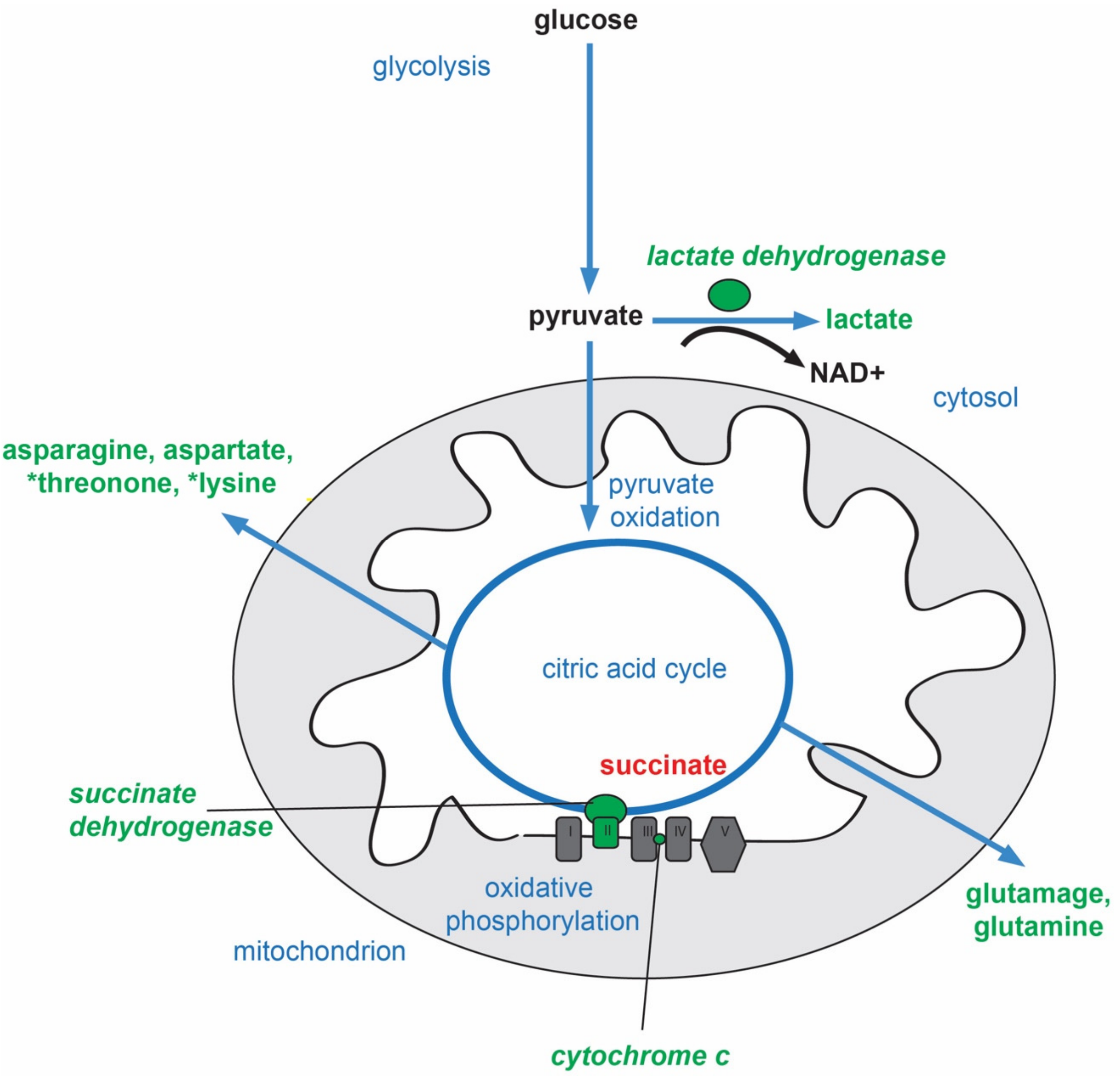

**References**

**Groot, A. P. de** (1952). Amino acid requirements for growth of the honeybee (Apis mellifica L.). *Experientia* **8**, 192–194.
